# Prospects of industrial consumption embedded final emissions: a revision on Chinese household embodied industrial emissions

**DOI:** 10.1038/s41598-020-58814-w

**Published:** 2020-02-04

**Authors:** Muhammad Jawad Sajid, Wanguan Qiao, Qingren Cao, Wei Kang

**Affiliations:** 1 School of Management, Xuzhou University of Technology, Xuzhou, Jiangsu China; 2 School of Economics and Management, Jiangsu Vocational Institute of Architectural Technology, Xuzhou, 221116 China; 3 School of Management, China University of Mining and Technology, Xuzhou, 221116 China; 4 Jiangsu Energy Economics and Management Research Base, Xuzhou, 221116 China

**Keywords:** Climate-change mitigation, Climate-change policy, Environmental impact

## Abstract

The final demand embedded emissions are mainly estimated by considering the intermediate industrial production. However, the industrial consumption embedded emissions are not well investigated. This study estimates both the industrial production and consumption embedded emissions of Chinese households. Our results indicate significant differences between household industrial production and consumption embedded emissions. These different patterns are due to the different set of emission multipliers, Leontief inverse vectors, and final pull effects employed in embedding the intermediate carbon consumption to final demand. “Electricity, Steam, Hot water production and supply” was the largest source of both urban and rural household’s industrial production embedded emissions. The largest amount of urban household’s industrial consumption embedded emissions was from ‘Miscellaneous intangible products’; while, for rural households it was ‘Food and Tobacco’. Shandong had the highest industrial embedded emissions from both approaches; however, the main sources of its embedded production and consumption emissions were different. This embedding of the intermediate industrial consumption emissions to household final demand provides new insights, for mitigating the household embedded carbon consumption. The uncertainty analysis indicated that sectors with bigger output values show higher uncertainty, and the input quantities of emission intensity and final demand were the main contributors to outcome uncertainties.

## Introduction

Given the fact that the household’s play a key role in industrial CO_2_ discharges as the final consumer of different type of goods and services, recently some nations have started considering the prospects of carbon mitigation from the perspective of household consumption^1^. Climate protection efforts are focused upon shifting the household consumption from high to low impact goods^2^. In fact, there are a number of ways in which households can reduce their carbon footprint^3^, but the correctly directed behaviour firstly requires the right targeting^4^. For Chinese low carbon development, it is quite vital to estimate different aspects of its households CO_2_ consumption^5^. During the decade of 1992–2002 Chinese household consumption greatly increased the industrial production, thus resulting in increased CO_2_ emissions offsetting the technological advancements achieved during this decade^6^.

The environmental extension of the famous Input-output model presented by Wisely Leontief^7^ has been extensively used to determine the embodied emissions in final demand. The model embeds the emissions from the industrial production processes to different categories of final demand. The household consumption related indirect carbon emissions can be ‘redefined’ and ‘recalculated’ by employing the input-output model^8^. Recently, some studies^1,9^ used the input-output model considering the capital formation as a productive industry, estimated the direct and embodied emissions of the Chinese urban and rural household consumption; these works state that it’s very important to correctly guide Chinese household consumption behaviour in order to achieve a cost-efficient reduction of industrial carbon emissions. There are also various other studies which have employed the usual input-output model (treating capital formation as a final demand category) to establish various facts about Chinese household embodied industrial carbon emissions. For example, that the indirect (embedded) carbon emissions are the main source of Chinese households carbon discharges^10^; there exists a huge gap between urban very rich and rural and urban poor carbon footprints, and that a shift in household choices has substantial climate improvement paybacks, tapping these potentials require developing carefully considered policy options and corresponding infrastructure resources^11^. Some further literature on Chinese household’s carbon emissions under the IO model is summarized in the following Table 1.

**Table 1:** Literature summary of Chinese household carbon emissions.

Study Period	Reference	Models	Main Findings
2007 and 2012	^28^	Semi-closed IO model and Hypothetical extraction method	This study considered household as an intermediate production industry; and estimated the impact of household consumption on provincial and inter-sectoral CO_2_ emissions of China. Found serious inequality of emissions between different household income groups and amongst rural and urban households.
1995–2009	^8^	IO, SDA and ‘multivariate regression analysis’	The sustained consumption of secondary sectors and the rapid increase in the use of territory industry products results in a constant increase in Chinese household’s indirect carbon emissions.
2007 and 2012	^29^	Semi-closed IO model and Hypothetical extraction method	This study, again by considering the Chinese household as a production sector, calculated the impact of different household income groups on China’s industrial carbon emissions. As per their results, urban household indirect carbon emissions have a much greater impact compared to the rural households; specifically, the extraction of urban highest income group causes the biggest relative reduction in CO_2_ emissions.
1992–2005	^61^	IO model and SDA	The increase in household consumption level was the key player in increasing household’s indirect CO_2_ emissions during the selected study period. For carbon mitigation, the Chinese government should streamline the economy and increase efficiency rather than reducing the consumption of goods.
2002, 2005, 2007, and 2010	^62^	IO model	The article made a comparative analysis between energy-related indirect household emissions from the China and the USA. Although, USA’s embedded household emissions were greater than China’s, over the years these emissions showed a downward trend, while Chinese household indirect emissions increased rapidly over time.
2002–2012	^63^	IO model and LMDI	Estimated the indirect CO_2_ emissions, their drivers and the income-based inequality effect on these carbon emissions, from Chinese urban households. The richest income group comprising 10% of the total, induced 21% of the indirect carbon emissions.

IO stands for ‘input-output’; SDA stands for ‘Structural decomposition analysis’, and LMDI is the abbreviated form of ‘logarithmic mean Divisia index’.

For considerable reductions to CO_2_ emissions, it is vital to think out of the box and search for new prospects to carbon emissions mitigation^12^. The problem with the current embedded final demand related literature (specifically for households) is that it embodies the total intermediate industrial production emissions to the final demand. In the current model, all carbon emissions from the production of goods and services generated during a specific time period are transferred to the final consumption^13^. However, this approach is limited to merely considering the emission intensity of the production industry whose final (household) embodied emissions are being estimated. Whereas, the consideration of final household embodied emissions from the intermediate intra plus inter-industrial consumption takes in to account not only the production sector emission intensity but also the emission intensities of its entire upstream supply chain. Furthermore, a recent study indicated more cost-efficient mitigation potential for China’s industrial carbon emissions considering industrial consumption rather than production of CO_2_ emissions (especially inter-sectoral carbon purchases); the study pointed out that focusing on high direct producers (emitters) of industrial carbon emissions often result in poor performances of carbon consuming (importing) sectors, this consequences in inter-sectoral carbon leakages within an economy^14^.

Final demand is ultimately responsible for the industrial emissions^15^; targeting final demand’s carbon consumption is often considered as a just and efficient measure to reduce emissions from industrial production^16^. Similarly, the considering of final embodied emissions from intermediate carbon consumption can help in reducing the carbon emissions from the industrial consumption. This approach by decreasing or restructuring the final demand of high carbon consuming industries (through modified carbon labelling, taxation, etc.) can help in achieving more just and cost-efficient carbon mitigation of the entire economy. In order to embed the discharges from intermediate industrial consumption to household’s final consumption, firstly we have to estimate the intermediate industrial carbon linkages. The modified hypothetical extraction method (MHEM)^17^ has been widely used to calculate intermediate industrial carbon linkages. Under the MHEM the industrial carbon linkages are decomposed in to net forward (sales), net backward (purchases), internal (intra-sectoral) and the mixed (re-purchases) intermediate industrial CO_2_ linkages. Explicitly, the model has been employed to estimate inter-sectoral^14,18–23^ and interregional^24,25^ industrial carbon and pollutant linkages. Here, the MHEM has been modified to estimate urban and rural household embodied emissions from inter and intra-sectoral consumption.

The article draws a significant comparison between the conventional Chinese national and regional (provincial) household embedded emissions from industrial production and the unconventional embedded emissions from industrial consumption and reports findings which can direct policymakers in devising effective carbon relabeling of the world’s topmost emitter’s household embedded intermediate industrial emissions and thus providing out of the box solutions to achieve direct and indirect industrial carbon mitigation, which are a major cause of global climate change. Finally, uncertainty measurement evaluation using ‘GUM’ propagation of uncertainty and the ‘Monte Carlo simulation’ approaches is also presented, the uncertainness in sum of provincial accounts are measured against the standard national accounts. It has been reported that the provincial energy statistics of the official yearbooks (used as basis for the carbon intensity measurements in this study) contain some issues, for example there exists huge margin between combined sum of provincial and the national energy data^26^. Also, only a handful of studies using the input-output models have considered the uncertainty analysis of the measurements^27^. The studies using input-output model normally do not include the ‘uncertainty analysis’, thus the addition of this analysis further add to the novelty of our paper.

## Results

### Carbon emissions from the total and household induced inter and intra-sectoral consumption

The household embedded inter-sectoral carbon consumption accounted for almost 24% of the total inter-sectoral CO_2_ consumption over the period. Compared to total final demand embedded inter-sectoral carbon consumption, household embedded inter-sectoral CO_2_ consumption emissions grew more steadily over time. The highest share of total demand embedded inter-sectoral carbon consumption came from the Construction sector, which accounted for approximately 32% of emissions over time. These results are quite in line with the findings of other studies calculating the total backward industrial carbon linkages of Chinese economy, where they have consistently reported the Construction sector to be the prime source of Chinese inter-sectoral carbon imports^14,22^. Contrarily, the highest source of household embedded inter-sectoral consumption with approximately 20% came from the ‘Miscellaneous Intangible Products’ sector. Which is in contrast with the results of the studies reporting the household embedded industrial production emissions, where the electricity production usually embeds the highest amount of carbon emissions^10^.

**Figure 1:**
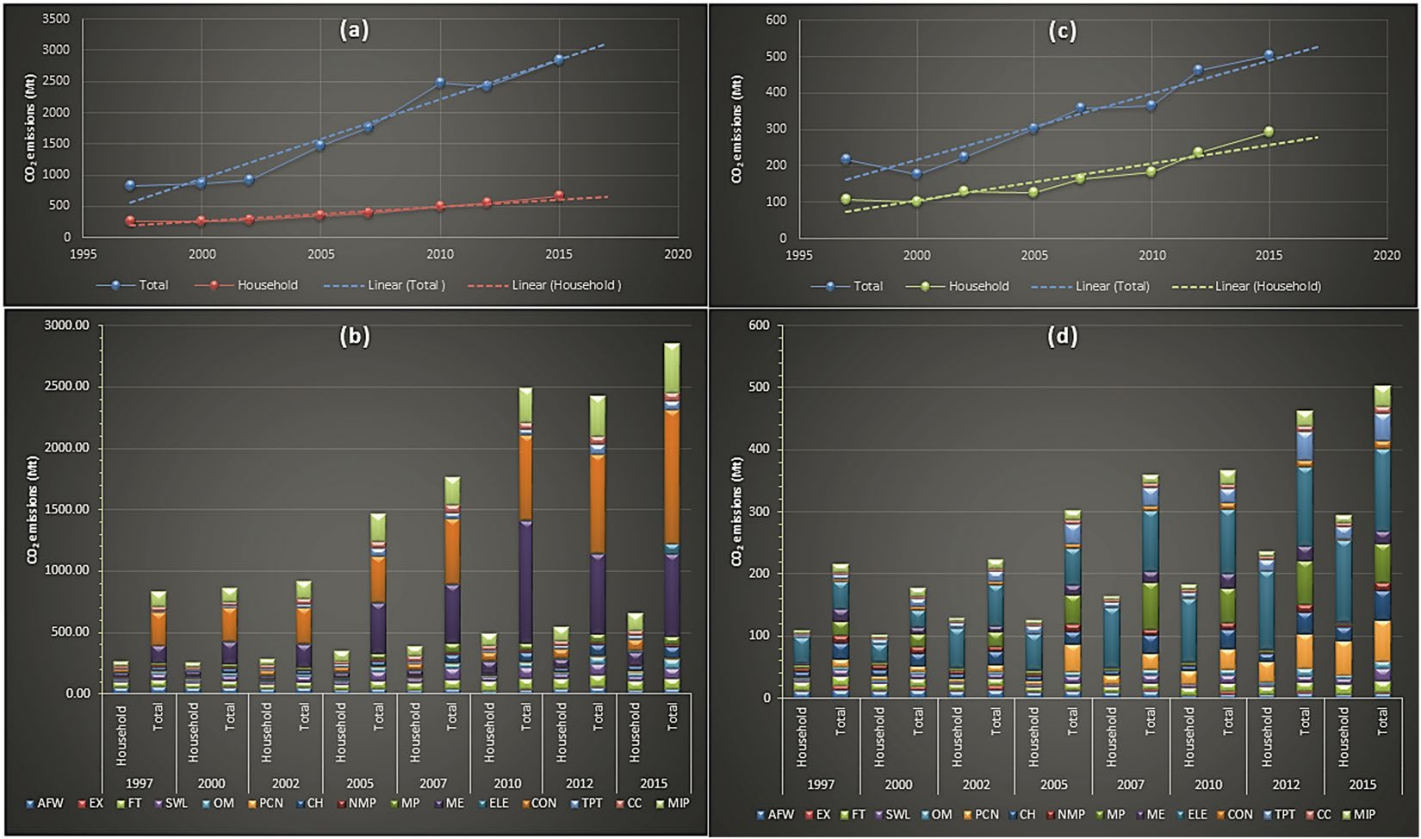
Comparison of results between total final demand and household embodied inter and intra-sectoral carbon emissions. **(a**,**b)** denote aggregated and sectoral decomposition of total final demand and household embedded temporal emissions from intermediate inter-sectoral consumption; and **(c**,**d)** denote sectoral decomposition of the total final and household embedded temporal emissions from intra-sectoral consumption respectively.

Both the total and household embodied intra-sectoral consumption emissions were considerably less than the total and household embedded inter-sectoral CO_2_ consumption. However, compared to the steady growth of household indirect emissions from the inter-sectoral carbon consumption; the household embedded intra-sectoral consumption grew sharply and moved very closely with the total intra-sectoral carbon demand. The household emissions from the intra-sectoral demand constitute approximately 51% of total emissions form the intra-sectoral consumption. The highest amount of both the total final demand and household embedded intra-sectoral emissions with approximately 25% and 48% respectively came from the ‘Electricity, Steam, Hot water production and supply’ sector. Supplementary Table S1 contains the details of the sectoral emissions intensities and of the sectoral coding used for the graphical presentations. Figure 1 shows the carbon emissions from the total and household induced inter and intra-sectoral consumption.

### Urban and rural households embodied carbon emissions from inter and intra-sectoral carbon consumption

**Figure 2:**
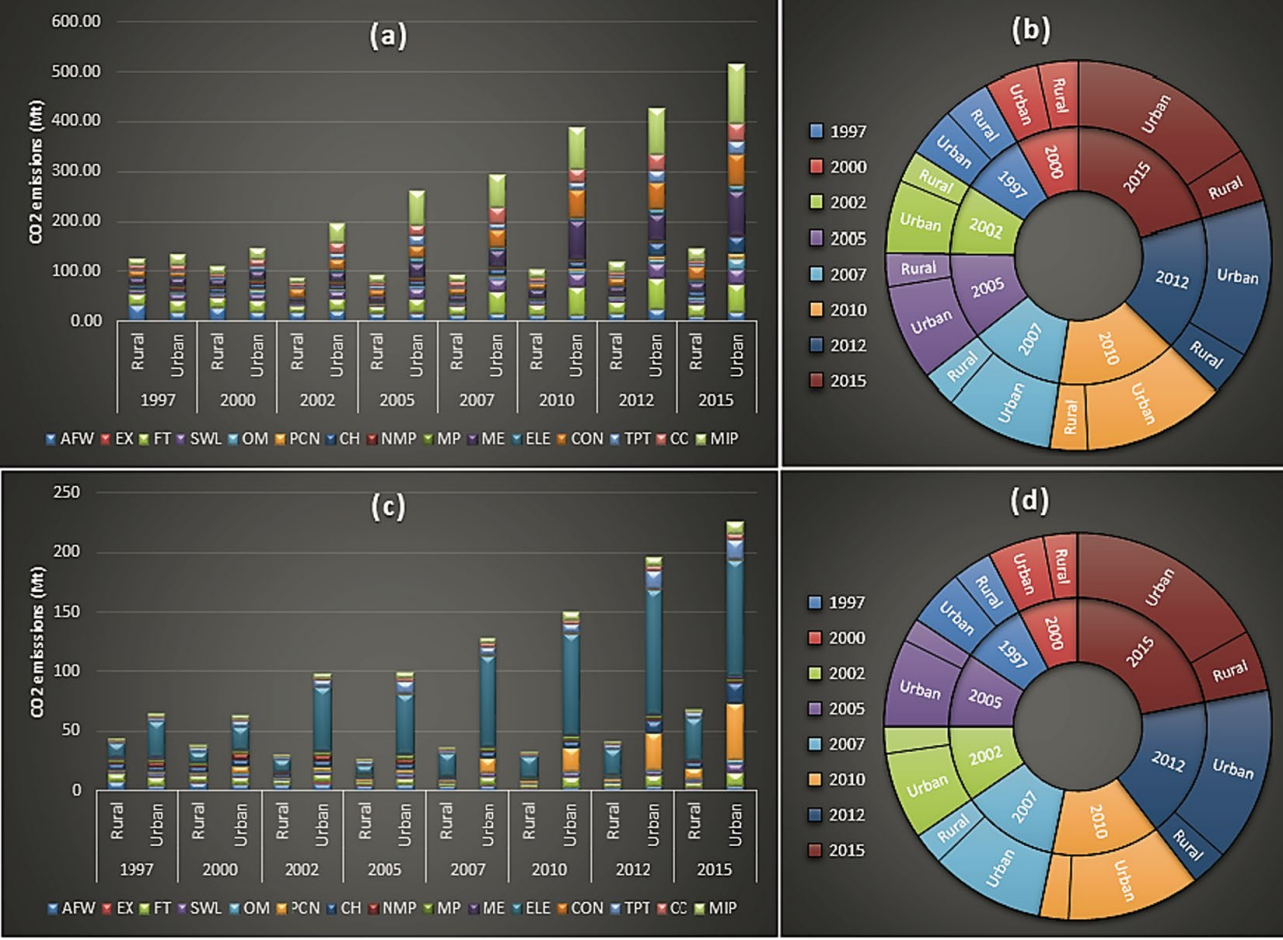
Comparative emissions between urban and rural household embodied emissions from inter and intra-sectoral intermediate consumption. **(a)** presents sectoral decomposition and **(b)** represents the aggregation of embodied urban and rural temporal carbon emissions from inter-sectoral consumption; **(c)** represents sectoral decomposition and **(d)** presents aggregation embodied urban and rural temporal carbon emissions from intra-sectoral consumption.

There was a huge gap concerning the China’s urban and rural household embodied emissions from inter and intra-sectoral carbon consumption. This is a well-established fact now, that the carbon footprint from the Chinese urban household consumption is far superior than that from its rural household consumption^1,9,11,28,29^. Urban household embodied inter-sectoral carbon consumption with a compound annual growth rate (CAGR) of approximately 18% increased sharply over time, while rural household embedded inter-sectoral CO_2_ consumption witness only a 2% growth rate. Similarly, Chinese urban household embedded intra-sectoral carbon consumption with a CAGR value of almost 17% grew much dramatically as compared to rural household growth of only 6%. The ‘Miscellaneous intangible products’ industry with approximately 22% share over time was the biggest source of urban household embodied inter-sectoral carbon consumption. Whereas, for China’s rural households ‘Food and Tobacco’ with approximately 17% was the largest source of embedded emissions from inter-sectoral CO_2_ use. Contrarily, the ‘Electricity, Steam, and Hot water production and supply’ sector with approximately 50% and 41% respectively was the largest source of both the urban and rural households embedded emissions from intra-sectoral industrial consumption. Figure 2 displays urban and rural households embodied carbon emissions from inter and intra-sectoral carbon consumption.

### Comparison between urban and rural final demand embedded emissions from intermediate industrial production and inter and intra-sectoral consumption

**Figure 3:**
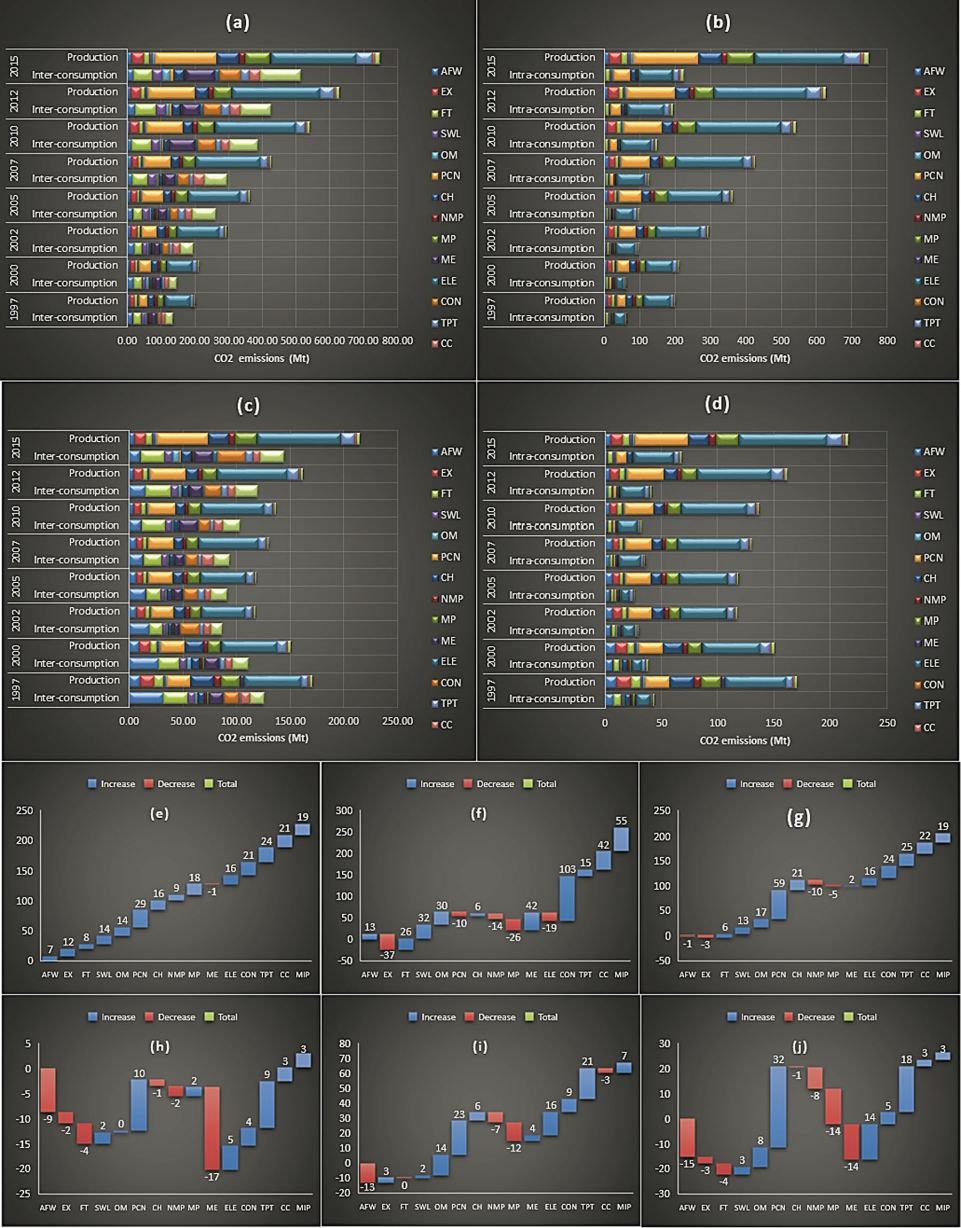
Comparative results of urban and rural household embedded emissions from industrial production and inter and intra-sectoral consumption, and compound annual growth rates. **(a**,**b)** represent comparison between urban household embedded emissions from production and inter and intra-sectoral consumption; **(c**,**d)** represent comparison between rural household embedded emissions from production and inter and intra-sectoral consumption; **(e–g)** present compound annual growth rates of urban household embodied emissions from industrial production, inter-sectoral and intra-sectoral consumption; **(h**–**j)** represent compound annual growth rates of rural household embodied emissions from industrial production, inter-sectoral and intra-sectoral consumption respectively.

There were quite considerable differences between the patterns of urban and rural household embedded emissions form inter plus intra-sectoral consumption, and the embodied emissions from the industrial production. The highest net increase in urban households embedded industrial production emissions with 29% was from the sector of ‘Petroleum processing, Coking and Nuclear fuel industry’. Contrarily, quite different patterns of absolute emissions values and net emissions increases can be seen from Fig. 3, for the Chinese urban household embedded emissions from the inter-sectoral carbon consumption. Construction’ industry with approximately 103% net emissions increase was at first place. On the other hand, ‘Machinery and Equipment’ who witnessed a net decrease of approximately −1% to the urban household embedded industrial production emissions, saw a 42% net increase to embodied inter-industrial consumption emissions. Urban households embodied intra-sectoral carbon consumption just as the industrial production embedded emissions saw the most drastic increase from the ‘Petroleum processing, Coking and Nuclear fuel industry’ having approximately 59% net emissions increase. But contrary to both the production and inter-sectoral industrial consumption embedded emissions, the urban households embodied emissions from intra-sectoral consumption witnessed a net decrease of −1% to the embedded emissions from the ‘Agriculture, Forestry, Animal Husbandry, Fisheries and Water Conservancy’.

The largest net increases towards rural households embodied emissions from production with approximately 10% was from ‘Petroleum processing, Coking and Nuclear fuel industry’. The sectors of ‘Machinery and Equipment’ with −17% saw the biggest net emissions decrease over the study period. Also, the biggest net increases to rural household embed inter-sectoral carbon consumption was from Petroleum processing, Coking and Nuclear fuel industry’; however, the embodied inter-sectoral carbon consumption from this industry showed different proportion of 23% increase. In contrast, sectors like ‘Extractive industry’ which saw net decreases of −2% in rural household embodied industrial production emissions, witnessed a positive net increases of 3% towards rural household embedded inter-sectoral consumption emissions. The biggest contrast to the net emissions over time between the rural household embedded intra-sectoral consumption emissions and the industrial production embedded emissions came from ‘Metal products manufacturing’ industry, where the former witnessed a net decrease of −14% while the latter saw a net increase of approximately 2% respectively.

### Comparison between Chinese household’s industrial production and consumption embedded emissions

The Chinese household industrial consumption (inter plus intra-sectoral) embodied emissions with a value of 4.57 billion tons were slightly less than the embedded emissions from the industrial production 4.63 billion tons over time. However, there were huge differences between the size of sector-wise embedded emissions from both the industrial production and consumption. For example, ‘Construction’ sector embodied industrial consumption emissions were at least 52 times higher than that from intermediate industrial production over time. While, the sector of ‘Extractive industry’ almost had 16 times higher household embedded industrial production emissions compared to consumption.

**Figure 4:**
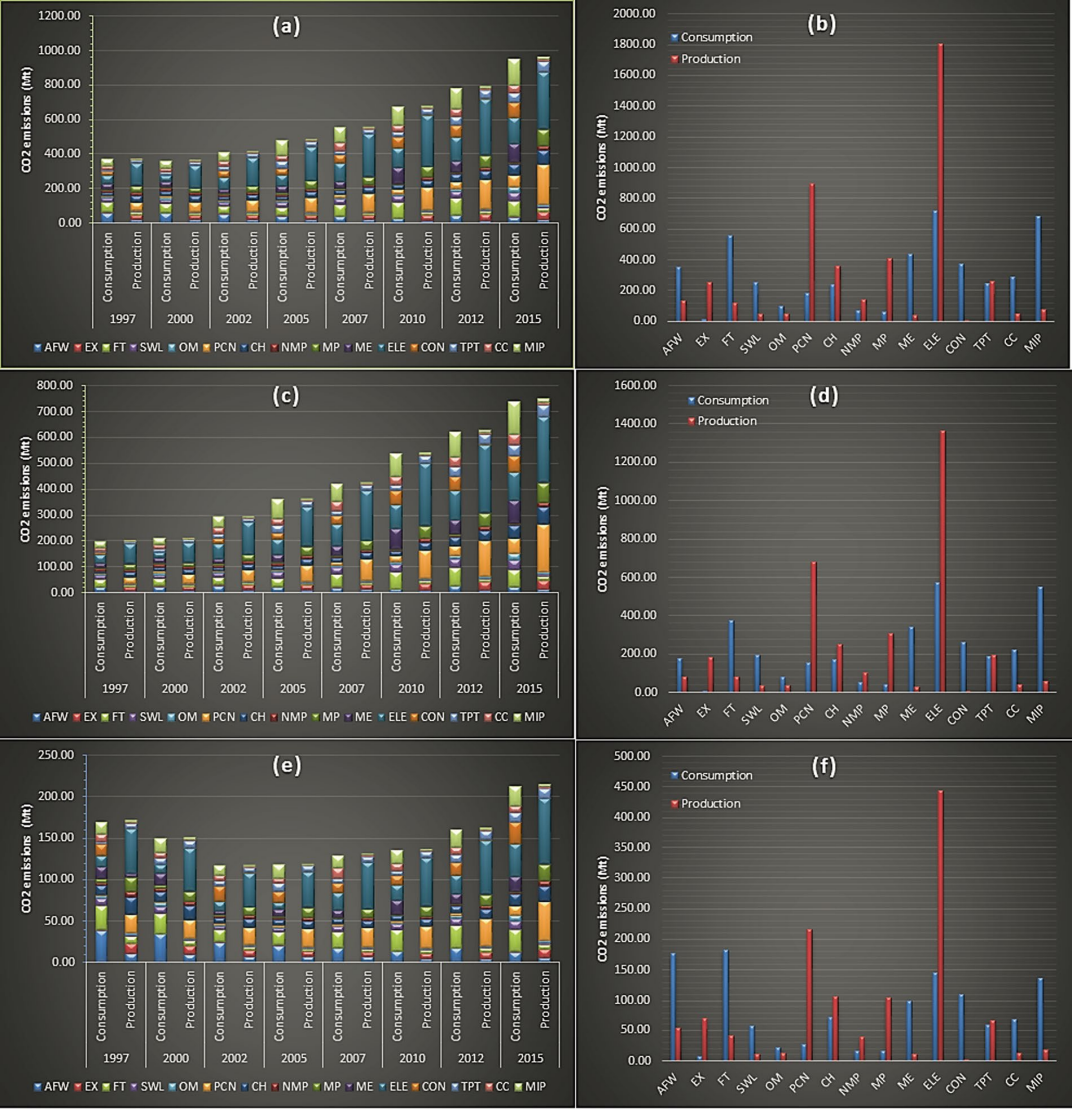
Comparative results of total, urban and rural household embodied emissions from intermediate industrial production and consumption. **(a**,**b)** represent decomposed and aggregated total household temporal embodied emissions from intermediate industrial production and consumption; **(b**,**c)** represent decomposed and aggregated urban household temporal embodied emissions from intermediate industrial production and consumption; **(e,f)** represent decomposed and aggregated rural household temporal embodied emissions from intermediate industrial production and consumption.

Urban household embedded emissions from industrial production and consumption were almost 3.42 and 3.38 billion tons over the years. And rural household embodied emissions from industrial production and consumption were approximately 1.20 and 1.19 billion tons over time. Just like household embodied emissions from industrial production and consumption, the embedded urban and rural household emissions also indicate large differences between these two types of emissions. The ‘Construction’ sector had 52 times larger embedded industrial consumption embedded from urban household compared to embedded emissions from intermediate industrial production; and for the rural households the industrial consumption embodied emissions from this sector were 52.3 times higher than that from intermediate industrial production. Alternatively, for the urban households, the intermediate production emissions from ‘Extractive industry’ were 23 times higher than from industrial consumption. Similarly, the rural households embedded production emissions from this sector were 10 times higher than from intermediate industrial consumption of goods and services. Figure 4 displays Chinese household’s industrial production and consumption embedded emissions.

### Chinese regional household embedded industrial production and consumption emissions

**Figure 5:**
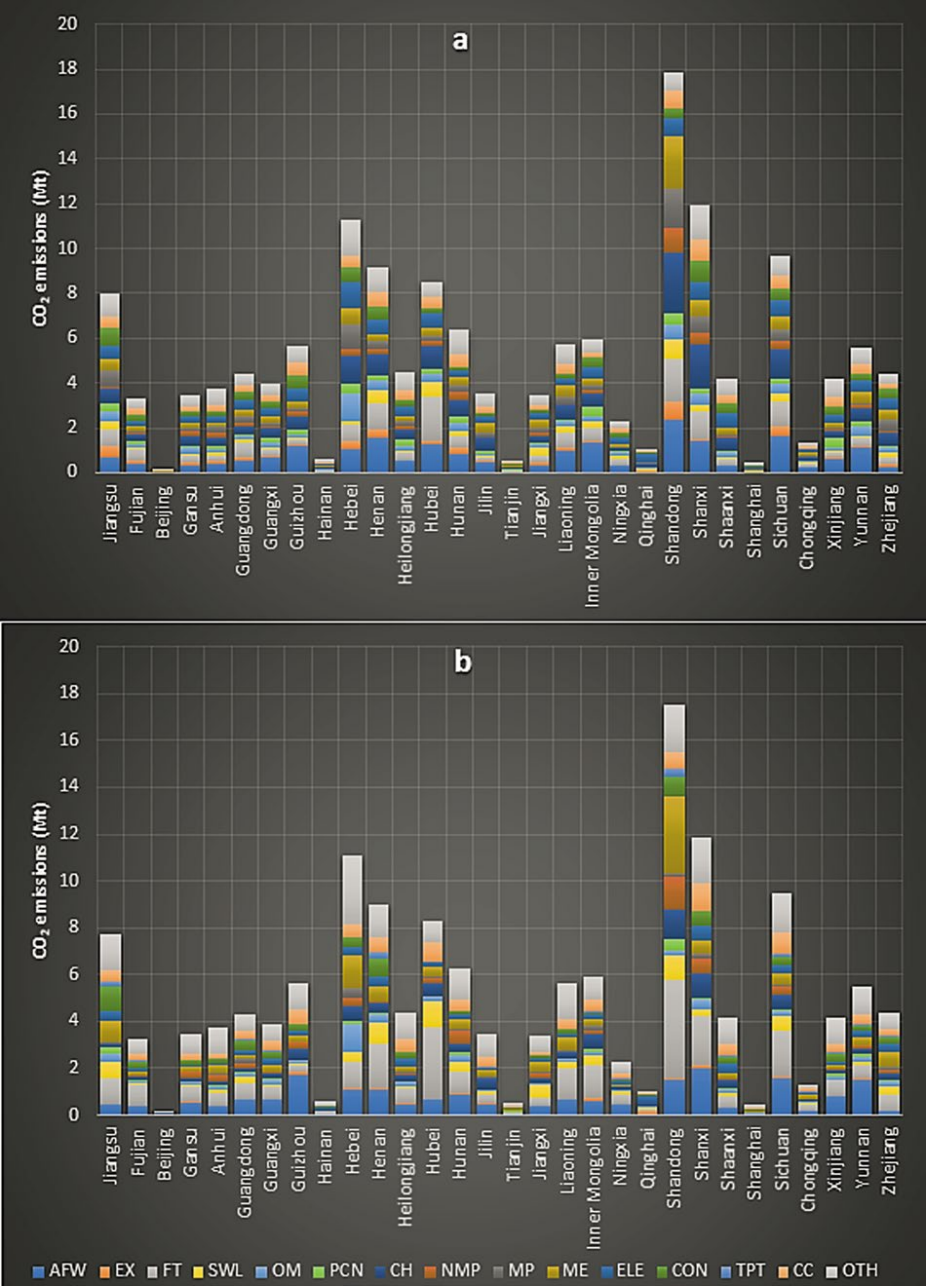
Rural household embedded regional industrial emissions; **(a)** industrial production **(b)** industrial consumption embedded regional emissions.

Chinese provincial plus federally operated municipalities’ rural household embedded industrial production and consumption emissions for the year 2012 were 155.18 and 152.64 Mt respectively. Shandong with 17.87 and 17.50 Mt was the largest source of Chinese rural industrial production and consumption regional carbon emissions. It was followed by Shanxi (11.95 and 11.83 Mt) and Hebei (11.25 and 11.10 Mt) respectively. However, there existed some differences between both the rural household embodied industrial production and consumption emissions. For example, largest source of Shandong’s rural household industrial production embedded emissions with 2.70 Mt was the ‘Chemical industry’. In contrast, the biggest source of its industrial consumption embedded carbon emissions with 4.16 Mt was the sector of ‘Food and Tobacco’. Figure 5, presents the industrial production and consumption embedded rural household regional carbon emissions.

**Figure 6:**
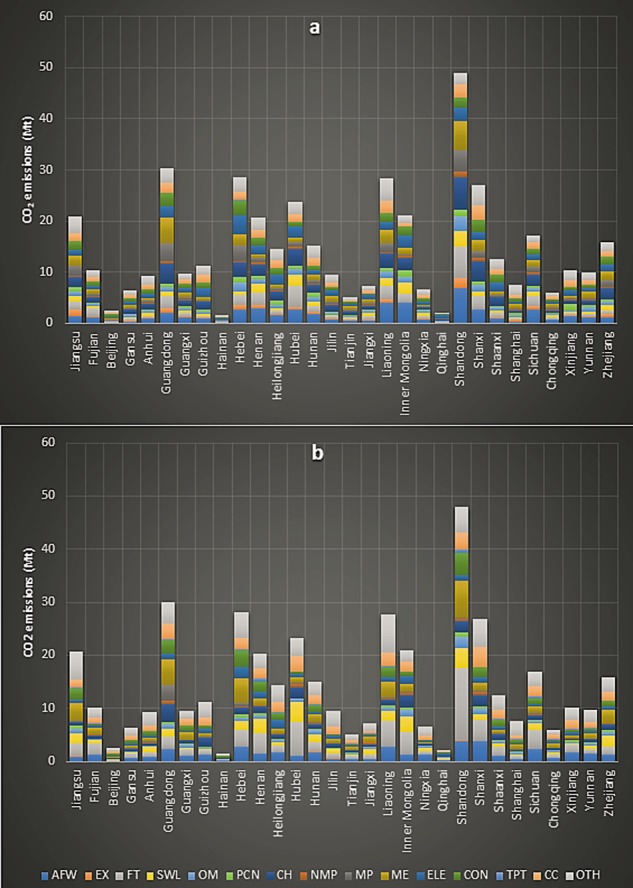
Urban household embedded regional industrial emissions; **(a)** industrial production **(b)** industrial consumption embedded regional emissions.

While, Chinese regional urban household industrial production and consumption embodied carbon emissions for 2012 were 441.13 and 434.79 Mt respectively. The highest source of both these two type of urban household embedded emissions with 48.93 and 48 Mt was again Shandong province. It was followed by Guangdong (30.38 and 30.02 Mt) and Hebei (28.48 and 28.07 Mt) respectively. However, the main industrial sources of these regional carbon emissions were quite different. For example, the highest source of Shandong’s urban household embedded industrial production emissions with 6.94 Mt was ‘Agriculture, Forestry, Animal Husbandry, Fisheries and Water Conservancy’, but the greatest source of industrial consumption embedded emissions with 13.47 Mt was ‘Food and Tobacco’. Figure 6, presents the industrial production and consumption embedded urban household regional carbon emissions.

**Figure 7:**
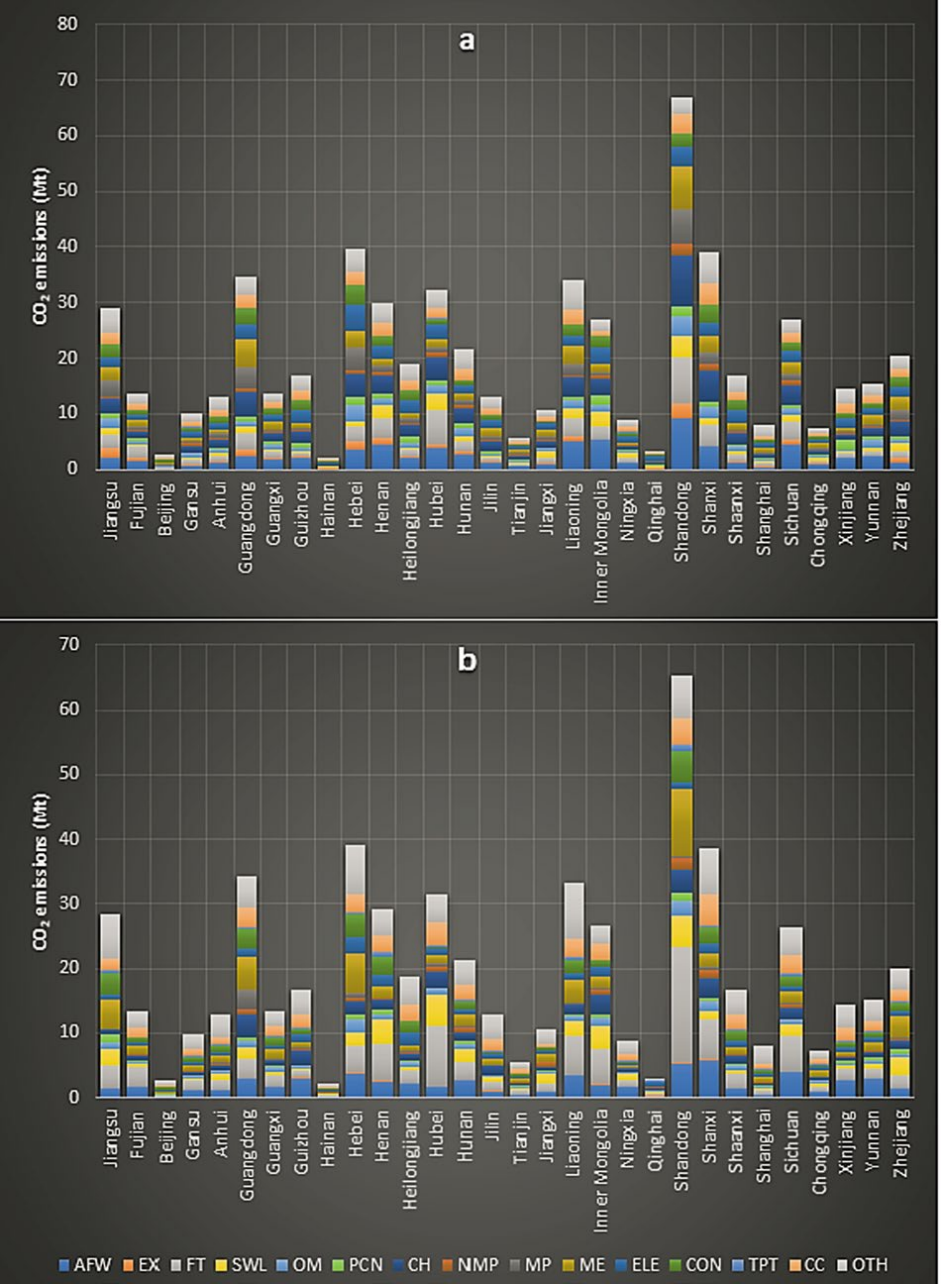
Total household embedded regional industrial emissions; **(a)** industrial production **(b)** industrial consumption embedded regional emissions.

Chinese household (urban plus rural) total industrial production and consumption embedded regional emissions for 2012 were 596.31 and 587.43 Mt respectively. Shandong with 66.80 and 65.49 Mt had the highest amount of both industrial production and consumption embedded household emissions. It was followed by Hebei (39.73 and 39.16 Mt) and Shanxi (39.08 and 38.67 Mt) respectively. However, Shandong’s highest amount of industrial production embedded emissions with 9.17 Mt came from ‘Chemical industry’, while the largest amount of industrial consumption embedded emissions with 17.63 Mt came from the sector of ‘Food and Tobacco’. Figure 7, presents the household industrial consumption and production embedded regional emissions of China.

### Uncertainty of the results based upon the provincial statistics

As mentioned above in the introduction section, that it has been reported that the Chinese provincial and national energy data did not correspond to each other. Furthermore, it is also interesting to measure the uncertainties arising for the differences in national and provincial I/O data, including the intermediate and the final demand. In this case, the national data statistics should be considered as the standard. In this section, we present the uncertainties related with the aggregated results obtained under the available provincial data and compared it with the results obtained from the standard (reference) national data. For uncertainty measurement purposes the values of the national data were considered as the expected values, the uncertainty of the provincial accounts from their expected national accounts are presented in this section in detail. The values obtained are presented in the same unit as the original outcome value i.e. millions of tons of carbon emissions.

The uncertainties results are estimated ‘according to GUM (JCGM 100:2008)’. The coverage factor is set at 2, which corresponds to 95% coverage probability or confidence interval. Because the category of all the input quantities is ‘B’ the effective degrees of freedom for the ‘GUM LPU’ method is infinite (∞). For the ‘Monte Carlo’ simulation, the number of trials was set at 100000, greater number of trials tend to give better results. The results from the conventional ‘GUM’ LPU and the ‘Monte Carlo’ simulation for rural, urban and total household embedded emissions are presented below. It should also be noted that, here for uncertainty estimations we only have considered the general model for embedding the industrial production embedded household emissions.

#### Sectoral uncertainties

**Figure 8:**
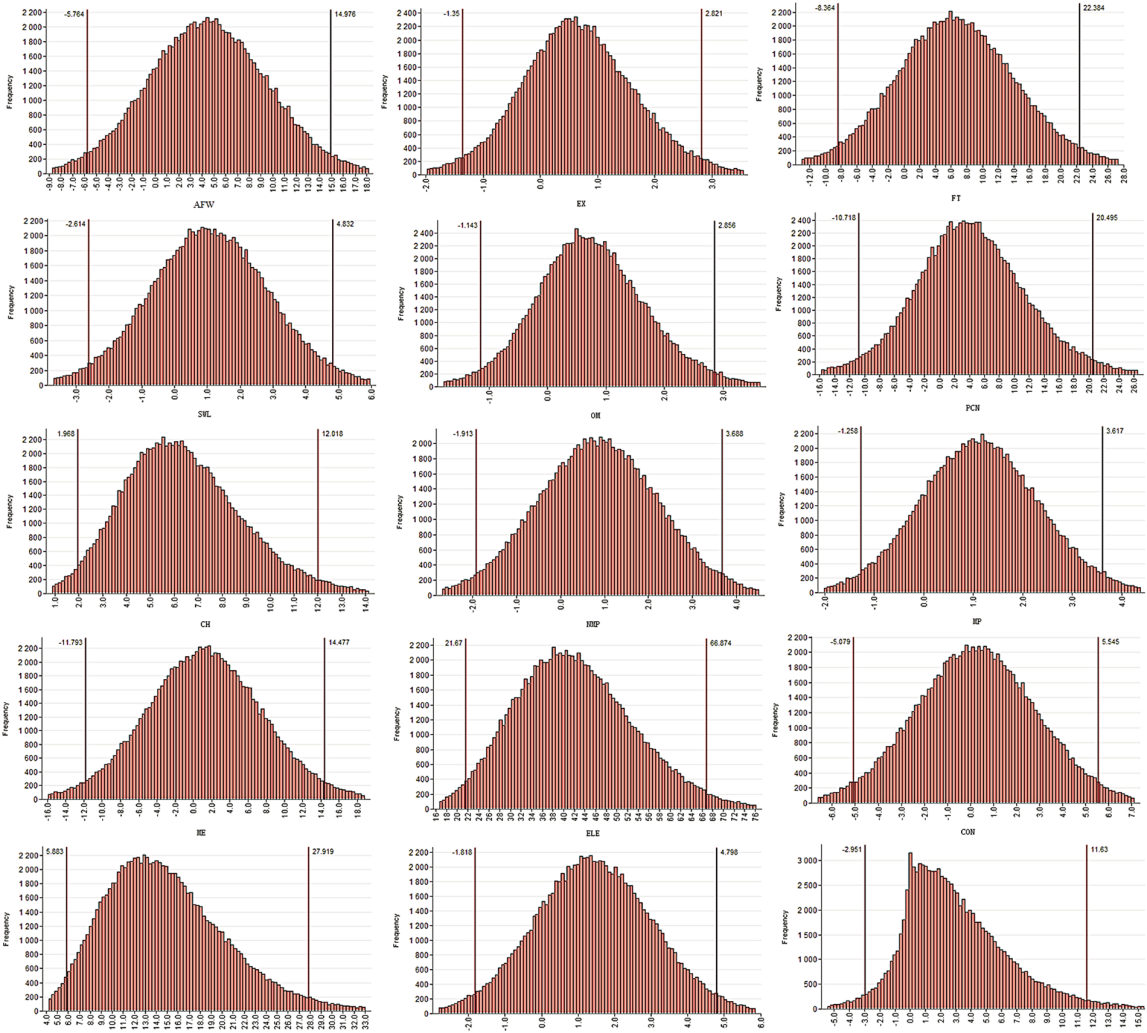
Rural households simulated data density of output values generated under the Monte Carlo method.

Supplementary Table S2 presents the sector wise uncertainties obtained under the conventional ‘GUM’ LPU method related with the estimation of uncertainties of rural household embedded emissions from the provincial data. While Supplementary Table S3 presents the results obtained from the Monte Carlo simulation approach. The results obtained including the value, combined standard uncertainty, and the expanded uncertainty are almost the same, although there are some exceptions with the values of the upper and the lower quantiles obtained under the two approaches. The highest combined standard and expanded uncertainties are associated with the sector of ‘Electricity and Steam, Hot water production and supply’ followed by the ‘Food and Tobacco’ and the ‘Petroleum processing, Coking and Nuclear fuel industry’ sectors. Figure 8, presents the simulated data density of output values generated under the Monte Carlo method.

**Figure 9:**
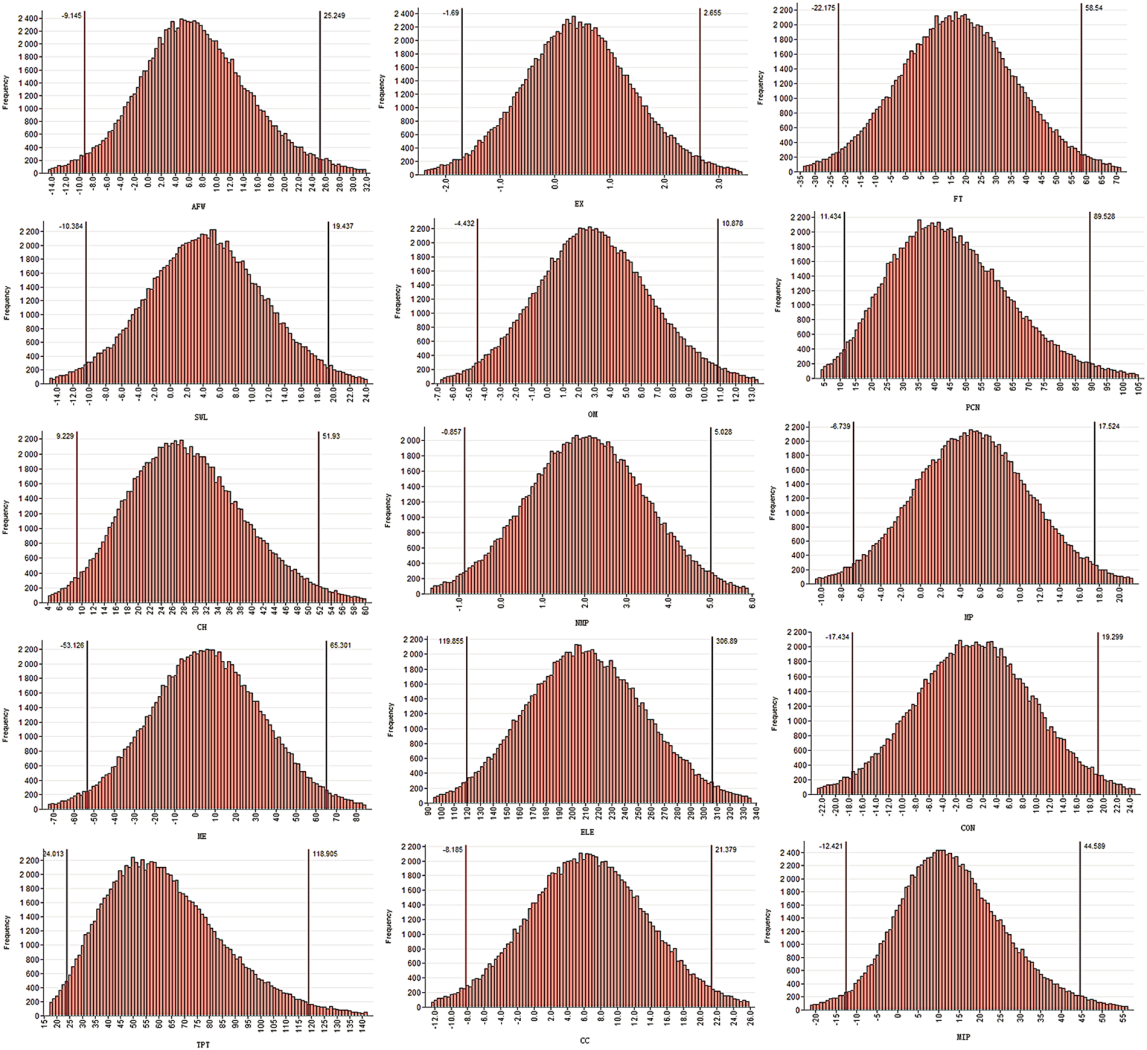
Urban households simulated data density of output values generated under the Monte Carlo method.

Tables S4 and S5 present the urban household embedded emissions estimate uncertainties based on the provincial data. Again, both the traditional GUM propagation and Monte Carlo simulation approaches almost yielded the same results of the uncertainties related to outcome value (urban household embedded industrial production emissions). The highest combined standard and expanded uncertainties were from the sector of ‘Electricity and Steam, Hot water production and supply’. It was followed by high combined standard and expanded uncertainties from the sectors of ‘Transport, Post and Telecommunication’ and ‘Machinery and Equipment’ respectively. Figure 9, presents the simulated data density of output values generated under the Monte Carlo method.

**Figure 10:**
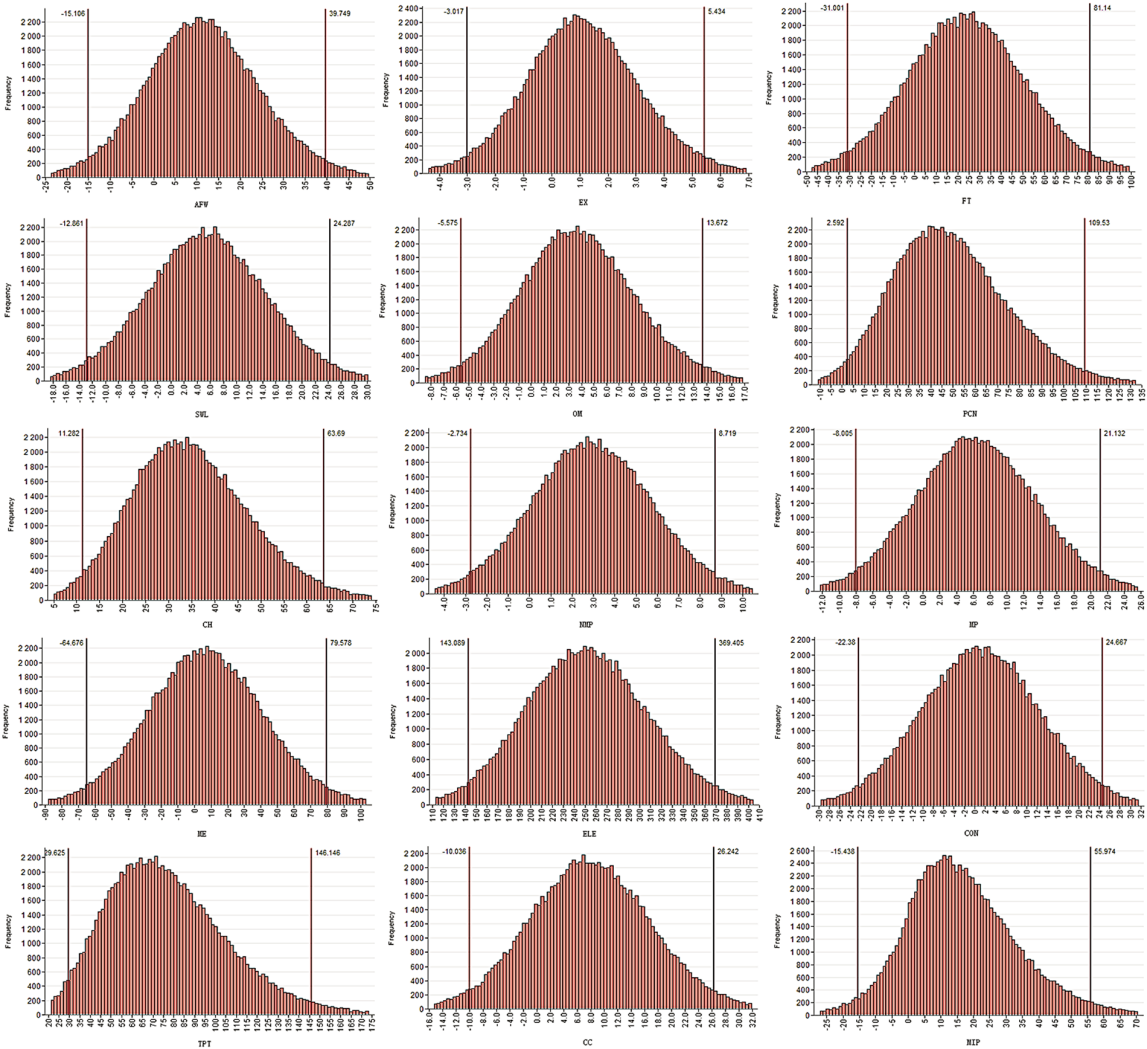
Total households simulated data density of output values generated under the Monte Carlo method.

Tables S6 and S7 present the sectoral combined standard uncertainties evaluated under the GUM and the Monte Carlo methods, including expanded uncertainties and lower and upper quantile values of the total household embedded emissions based on the provincial accounts. Again, ‘Electricity and Steam, Hot water production and supply’ had the highest combined standard and expanded uncertainty values under both the conventional GUM and the Monte Carlo approaches. The ‘Machinery and Equipment’ and the ‘Transport, Post and Telecommunication’ respectively followed it. Figure 10, presents the simulated data density of output values generated under the Monte Carlo method.

#### Uncertainty measurement of the different input quantities

Its, also quite vital to understand the combined standard uncertainty, sensitivity and the uncertainty contribution related to the input-output quantities of emission intensity, Leontief inverse and the final demand. Table S8 (rural household), Table S9 (urban household) and S10 (total household) presents the input quantities (parameters) related uncertainties measurements. Each input quantity’s contribution to the uncertainty of the sectoral emission outcome is calculated by taking a product of the combined standard uncertainty and sensitivity coefficient of that input for a particular sector. For the rural, urban and total household embedded carbon emissions, the highest amount of ‘combined standard uncertainty’ for almost all the sectors quite obviously due to larger values came from the final demand. However, the largest value of almost all of the sectors sensitivity came from the emission intensity. Furthermore, the highest amount of input quantities contribution to sectoral uncertainty with some exceptions also came from the input of emissions intensity, the emission intensity estimation is based upon the provincial energy data from the National Bureau of statistics of China. The exceptional industries like ‘Extractive industry’, ‘Petroleum processing, Coking and Nuclear fuel industry’, ‘Non-metallic Mineral Products’, ‘Metal products manufacturing’ and ‘Transport, Post and Telecommunication’, had the highest value of their ‘uncertainty contribution’ from their respective final demands.

## Discussion

China is the World’s largest carbon emitter. Chinese households have been an important factor contributing towards the indirect carbon emissions from the intermediate industrial production processes. Generally, compared to the rural households, the urban households have played more vital role in promoting the household induced industrial carbon emissions. This study proposes an alternative way for calculating the embodied carbon emissions from the intermediate industries. The model estimates the households embodied emissions from the intermediate industrial consumption rather than from intermediate industrial production. There were some interesting differences in the results obtained under the two approaches; these differences were mainly due to different procedures adopted for the calculation of embedded final emissions. It should be reminded that, embedding emissions to final demand under the input-output model requires three main input quantities these include: the direct emissions intensity vector, the Leontief inverse matrix, and the vector of final demand. Under the usual intermediate production embedded emissions model a sectors final embedded emissions are based on the: direct emission intensity of the production sector, Leontief row vector, and the final demand vector, where the sectors own final demand plus the final demand of other sectors are pulling the sectors intermediate production. While, the model based upon embedding the intermediate industrial consumption emissions to final demand, uses the sectors own and all other upstream sectors emission intensities, isolates the sector’s consumption column of the Leontief inverse matrix, and only considers a sector’s isolated own final demand pulling the intermediate consumption. Which also makes it a more just and accurate model for presenting the embedding of the final demand emissions. Because this model not only includes the emission intensity effects of the upstream industries but also takes in to account a sector’s isolated specific category of final demand pulling the intermediate industrial consumption.

1. The usual approach embedding the industrial production emission intensity to the final demand only considers the carbon emission intensity of the production sector of a particular product. The embedding of intermediate consumption emission to the final demand besides considering the sector’s own emission intensity, also considers the emissions intensities of its upstream trading partners while embedding the emissions from intermediate industrial consumption to its final demand. Which provides four dimensional interconnected mitigation impact, whereby the carbon chain of household embedded emissions is based upon its own consumption, the production technology, the emission intensity of the consumption product, and intensities of the products entire upstream supply chain. The argument is further supported by the study conducted by^14^, their findings indicated more cost effective mitigation potential considering Chinese intermediate industrial carbon consumers rather than industrial producers. The intermediate industrial carbon consumption is ultimately for satisfying the final demand (in our case household final demand); so in order to curb the carbon consumption from the intermediate industrial use, a renewed and redirected mitigation policy based upon the patterns of household embedded emissions from inter and intra-sectoral consumption is required. Our comparative analysis yielded some interesting results, which may act as founding stone for redirecting the future mitigation efforts towards reducing the indirect industrial emissions caused by Chinese household’s final consumption.
2. The construction sector was the biggest source of China’s total emissions from intermediate industrial consumption. However, for Chinese household the ‘Miscellaneous intangible products’ sector was the largest source of embedded emissions from inter-sectoral carbon consumption over the years. The Chinese population (particularly urban) has been rapidly improving living standard, modernizing and digitizing over the years, this has led to ever increasing demand of ‘intangible products’ like mobile applications, software’s, digital experiences, financial services, education and entrainment industry etc. As per McKinsey Global Institute, China with more than 40% of global transactions is the world’s biggest e-commerce marketplace, its mobile payment market has already surpassed the USA by 11 times^30^. Although, this sector had very low direct carbon intensity over the years, but the high-embodied impact of this sector indicates that its procurement is not as clean. The additional benefit of using embedded emissions from industrial consumption rather than production shows consumers the real hidden impact of their purchases. That is the sectors that are considered clean under normal circumstances (the commonly green sectors): like services or intangible products, actually are considerably affecting the environment through their carbon purchases to fulfill their consumer’s final demand. This can encourage consumers to see through the actual hidden emissions caused by their consumption at the beginning of the process. It can also pave the way to think out of the box solutions; as we all know the usual approaches have not been sufficient enough to achieve the IPCC’s old target of 2 °C, let alone the new 1.5 °C global average temperature target. The household embedded emissions from intra-sectoral carbon consumption were considerably less than from the inter-sectoral consumption. The usual “Electricity, Steam, Hot water production and supply” sector was the biggest source of total final demand and household embedded emissions from the intra-sectoral consumption over time.
3. Further decomposition of household’s inter-sectoral consumption embodied emissions showed that net embedded emissions from urban household’s inter and intra-sectoral demand increased considerably over time, while net increase to rural household embedded emissions from both the inter and intra-sectoral consumption was comparatively less. Again for urban household’s the ‘Intangible products’ industry was the biggest source of inter-sectoral consumption embedded emissions. But, for the rural household the largest amount of inter-sectoral consumption embedded emissions came from “Food and Tobacco”. Here final demand scale adjustments through propagating less wasteful and ecological consumption and improving the carbon intensities of input-providing industries (cleaner procurement) can help in reducing the amount of embedded emissions from inter-sectoral consumption. Various studies have noted the growing trend of wasteful food consumption by Chinese households, which of course comes with social and environmental ramifications^31,32^. Furthermore, it has also been identified that there is a lack of sufficient public awareness in this regard^33^. For both the urban and rural households the biggest source of embedded emissions from intra-sectoral consumption was ‘Electricity, Steam, Hot water production and supply’. Here encouragement of energy saving habits and home-based renewable electricity generation options like solar PV and small wind turbines can help reduce the demand of the grid connected electricity. Furthermore, direct carbon intensity of this sector needs to be improved. Despite massive gains towards renewable energy production; still in 2017, almost 55% of China’s total electricity wad generated from the coal power^34^. Besides, increasing the share of renewable energy, the carbon emissions from coal power plants could be minimized by adopting to promising innovations like the carbon capture and storage.
4. There existed a huge difference amongst emissions patterns from the usual household embedded emissions from intermediate industrial production and the embedded emissions from inter-sectoral consumption. For example, the sector of ‘Extractive industry’; ‘Metal products manufacturing’; and ‘Electricity, Steam, and Hot water production and supply’; had quite considerable net increases of the embedded urban household emissions from industrial production, but contrarily experienced negative net emissions increases to inter-sectoral consumption embedded emissions. For rural households, ‘Extractive industry’; ‘Chemical industry’; and ‘Machinery and Equipment’ witnessed net emission decreases to embodied emissions from industrial production, while saw positive net increases to the inter-sectoral consumption emissions. For both urban and rural households there were also quite a few differences between net embodied emission increases from industrial production and intra-sectoral consumption. There was also huge difference in total, urban, and rural household embedded emissions from the intermediate industrial consumption (inter plus intra-sectoral) and the embodied emissions from intermediate industrial production. Although the total value of both the intermediate production and consumption embedded household emissions was fairly close, but vastly different patterns of Chinese total, urban, and rural households embodied emissions were detected between sector-wise intermediate industrial production and consumption embedded household emissions. All these figures indicate that there is need of re-carbon labelling of the embedded emissions from household consumption of industrial products specifically for the Chinese households. This renewed carbon labeling by targeting final demand induced industrial carbon consumption will not only be helpful in improving the household final consumption structure of high intermediate carbon consuming industries. Furthermore, it will also encourage the key intermediate carbon consumers in: improving the production technology; looking for alternative cleaner procurement options (including for energy and transport); encouraging and forcing (where feasible) their high intensity upstream selling partners to improve their respective carbon intensities; invest and collaborate in carbon efficiency projects of their upstream suppliers. Because the relabeled household embodied emissions based upon intermediate industrial consumption alongside with necessary legal amendments (carbon taxation, personal carbon trading) and public awareness projects (social media, electronic media and press) will require the households to reconsider their consumption of the high emissions embedded products. This will give rise to the four dimensional mitigation effect.
5. At provincial level, the Shandong province was the biggest source of both the production and consumption embedded total, rural and urban household embedded emissions. Other studies have also indicated Shandong to be a leading source of Chinese household embedded emissions^28^. However, there were differences between the industrial distributions of emissions between the two models. This indicates that the industrial production and consumption-embedded models actually do not much affect the total amount of industrial emissions of any particular region. Instead, the main difference between the two models lies in their allocation of industrial emissions to the final demand. That is the distribution of industrial emissions to different industries final demand is different under the two approaches.
6. Scholars’ give much importance towards the sources of data towards the I/O related studies. In fact, mostly the data released by the official statistical departments is preferred over the data gathered through different non-survey methods. Most governmental departments only provide single regional input-output tables, implying that the construction of the ‘multi-regional’ models usually depends upon the scientific community^35^. To save time and money the multi-regional input-output tables are usually constructed based on the ‘non-survey’ approaches, hence these tables are prone to errors and biasness^35^. This study also used the single regional input-output and the energy data from the official national statistical departments, which adds to the reliability of the data sources. Furthermore, studies have empirically shown that in case of single regional I/O model, the aggregation only affects the ‘internal’ carbon share amongst industries, and between the local production and imports^36^. The absolute (total) amount of carbon responsibility and production emissions does not change, because under the ‘single-regional’ model the total amount of distributed carbon emissions remains the same^36^. For the I/O data to be consistent with the available energy data, the authors aggregated the sectors as per ‘Chinese national economic industry classification standard GB/T 4754–2017’. This shows that uncertainties related to the sources of data related to national estimates reported in this study are not a topic of much concern. Furthermore, the sectoral aggregation will not affect the absolute value of national industrial emissions. Due to these facts, the limited amount of literature available on the topic of uncertainties measurements of the emission estimates under the I/O model, is mostly focused towards the uncertainties associated with the application of multi-regional I/O tables and emission factors (see for example^27,36,37^).
7. However, energy consumption or relocation of consumption in each sub-section is associated with high uncertainties and subjective justification. Particularly, for energy consumption data, it is often mentioned that the China’s national energy consumption data is not equal to the sum of provincial energy consumption data^26^. In general, there are some differences between the national data statistics and the statistics of each province. The common practice in the literature is that, when the whole country is taken as the research goal, only the data with unified national caliber will be used. However, when the research unit is each province, and the data imbalance appears, the national data will be used as the standard, and the actual data of each province will be used as the proportion, in order to achieve the consistency between national data and provincial data. In this study however the authors have preferred not to take the proportional provincial data. The reason is that, the authors wanted to estimate the measurement uncertainties of the sum of results obtained from raw provincial data by taking the national industrial emissions and the I/O data as the standard (reference) data.
8. In most of the cases the sector’s with higher outcome values (final embedded emissions) had the largest ‘combined standard uncertainty’ and the ‘expanded uncertainty’ values but there were some exceptions to this rule. Nevertheless, the values of both these measures of uncertainties indicated strong correlations with the size of the outcome values. Which indicates that in general it is better to employ the most possible disaggregated industrial energy/emission, and corresponding input-output accounts. As mentioned above, in single regional models it will not affect the overall value of the outcome, but it will only change the distribution of emissions amongst the different industries of an economy. Other studies (based on multi-regional I/O model) also exerted the importance of using the disaggregated data where possible^36^. Input-quantities related uncertainties analysis conducted under the GUM (JCGM 100:2008) approach indicated that the final demand had the largest value of ‘combined standard uncertainty’, which may be mainly because of the relatively large size of the final demand compared to other I/O quantities. In this case it’s better to use an input quantity’s ‘uncertainty contribution’ value to indicate it contribution to uncertainly of outcome value of a sector. Calculated as the product of the sensitivity coefficient and the ‘combined standard uncertainty’. Ten out of fifteen sectors have the highest contribution to uncertainty from the input quantity of the emission intensity. Remaining five had the highest contribution from the rural, urban and the total household’s final demand. This exerts that it is important that both the provincial energy yearly statistics and the final demand data to be reconciled with the corresponding national data of China.

## Methods

### Calculation of fossil-fuel-based direct total and per unit carbon emissions

This paper calculates the carbon emission coefficient by multiplying the average low calorific value (MJ/kg) of all kinds of energy by the carbon content per unit calorific value (t/TJ). The average low calorific value of all kinds of energy comes from China Energy Statistics Yearbook 2012 and 2016, and the carbon content per unit calorific value comes from the Guidelines for Preparing Provincial Greenhouse Gas Inventories^38^. This study for the purpose of estimating Chinese industrial energy-related emissions considers industrial consumption of eight major fossil fuel types including Coal, Coke, Gasoline, Kerosene, Crude Oil, Diesel Oil, Fuel Oil and Natural Gas.

1 $${\rm{C}}=\mathop{\sum }\limits_{k=1}^{8}{\rho }_{ik}{v}_{k}$$

Where C denotes the total direct industrial carbon emissions of the Chinese economy. ${\rho }_{ik}$ represents the K type fossil-fuel consumption of sector *i*, *v*_*k*_ represents the carbon emission factor value of K type fossil fuel. Correspondingly, the direct per unit carbon emissions (Carbon intensity) is obtained by dividing the total emissions by the vector of total industrial output.

2 $${\tau }_{i}=\frac{{C}_{i}}{{X}_{i}}$$

Where ${\tau }_{i}$ represents sector-wise fossil fuel-related direct CO_2_ emissions intensity; *C*_*i*_ represents sector-wise direct carbon emissions and *X*_*i*_ presents the total sectoral output.

### Embodied household emissions from the intermediate industrial production

The basic equation for the famous Leontief^7^ input-output model is presented as:

3 $$X={(I-A)}^{-1}D$$

Here *X* is the vector of the total output, $L={(I-A)}^{-1}$ is known as the Leontief inverse matrix, where *I* denotes a fitting identity matrix and *A* represents technology or intermediate demand matrix, whose element $\frac{{a}_{ij}}{{X}_{j}}$ presents the quantity required from the sector *i* for the production of one unit of product at the sector *j*. *D* represents the vector of final demand.

Keeping in mind the production-consumption relationship from the equation number three; the total output required to produce the final household demand *H* is presented through the following equation.

4 $${X}^{H}={(I-A)}^{-1}H$$

By multiplying the diagonalized direct emission intensity vector *τ* with the equation number four, we can have the embodied industrial production emissions form final household consumption.

5 $${C}^{H}=\tau {(I-A)}^{-1}H$$

The above equation can easily be decomposed in to the sector-wise production embodied emissions from the rural and urban household demand.

6 $${C}_{im}^{H}=\mathop{\sum }\limits_{i=1}^{j}\,\mathop{\sum }\limits_{m=1}^{n}{\tau }_{i}{(I-A)}^{-1}{H}_{im}$$

Where ${C}_{im}^{H}$ presents sector-wise household induced carbon emissions from the industrial production of goods and services, ${H}_{im}$ represents *m* type household consumption of sector *i*.

### Embodied household emissions from the intermediate industrial consumption

The hypothetical extraction method (HEM) creates a hypothetical economy where a specific target sector is deleted from an economy, after that it makes a comparison between the original and the hypothetical economies^18,20,39^.

For the purpose of estimating embodied household emissions from the intermediate industrial consumption and to easily understand the intermediate industrial structure we have disintegrated the Chinese economy in to the target sector ${\Omega }_{s}$ and remaining sectors of the economy $\,{\Omega }_{-s}$. The resultant economy $\Omega$ is presented below.

7 $$\Omega =[\begin{array}{cc}{\Omega }_{s,s} & {\Omega }_{s,-s}\\ {\Omega }_{-s,s} & {\Omega }_{s,s}\end{array}]$$

The modified hypothetical extraction method^17^ is based upon the Cella hypothetical extraction model^40^, under Cella’s model, the impact of target sector’s inter-sectoral purchases (imports) and sales (exports) is measured through extracting these linkages from an economic system. As the purpose of the original model including its environmental extension is generally to calculate intermediate industrial carbon linkages (see^21^); it does not distinguish between induced effects (emissions) from different categories of final demand. Likewise, the MHEM^17^ and its environmental extensions (see^14,18^) also do not disintegrate final demand in to its different categories.

The decomposed structure of total household embodied emissions from the intermediate industrial production is presented below:

8 $${C}^{H}=[\begin{array}{c}{C}_{s}^{H}\\ {C}_{-s}^{H}\end{array}]=[\begin{array}{cc}{\tau }_{s} & 0\\ 0 & {\tau }_{-s}\end{array}][\begin{array}{cc}{(I-{A}_{s,s})}^{-1} & {(I-{A}_{s,-s})}^{-1}\\ {(I-{A}_{-s,s})}^{-1} & {(I-{A}_{-s,-s})}^{-1}\end{array}][\begin{array}{c}{H}_{s}\\ {H}_{-s}\end{array}]=[\begin{array}{cc}{\tau }_{s} & 0\\ 0 & {\tau }_{-s}\end{array}][\begin{array}{cc}{\psi }_{s,s} & {\psi }_{s,-s}\\ {\psi }_{-s,s} & {\psi }_{-s,-s}\end{array}][\begin{array}{c}{H}_{s}\\ {H}_{-s}\end{array}]$$

Where $${C}^{H}=[\begin{array}{c}{C}_{s}^{H}\\ {C}_{-s}^{H}\end{array}]$$ presents the embodied household emissions for the target and rest of the sectors, $$[\begin{array}{cc}{\tau }_{s} & 0\\ 0 & {\tau }_{-s}\end{array}]$$ presents the direct emission intensities, $$L=[\begin{array}{cc}{\psi }_{s,s} & {\psi }_{s,-s}\\ {\psi }_{-s,s} & {\psi }_{-s,-s}\end{array}]$$ is the Leontief inverse matrix, and the household final demand is equal to $$[\begin{array}{c}{H}_{s}\\ {H}_{-s}\end{array}]$$.

Under the Cella^40^ model, the target sector does not purchase or sell to other sectors of an economy, the equation presenting the extraction of target sector’s household induced carbon purchases and sales is described below:

9 $${C}^{H\ast }=[\begin{array}{c}{C}_{s}^{H\ast }\\ {C}_{-s}^{H\ast }\end{array}]=[\begin{array}{cc}{\tau }_{s} & 0\\ 0 & {\tau }_{-s}\end{array}][\begin{array}{cc}{(I-{A}_{s,s})}^{-1} & 0\\ 0 & {(I-{A}_{-s,-s})}^{-1}\end{array}][\begin{array}{c}{H}_{s}\\ {H}_{-s}\end{array}]=[\begin{array}{cc}{\tau }_{s} & 0\\ 0 & {\tau }_{-s}\end{array}][\begin{array}{cc}{\psi }_{s,s} & 0\\ 0 & {\psi }_{-s,-s}\end{array}][\begin{array}{c}{H}_{s}\\ {H}_{-s}\end{array}]$$

Where $${C}^{H\ast }=[\begin{array}{c}{C}_{s}^{H\ast }\\ {C}_{-s}^{H\ast }\end{array}]$$ represents the household final demand embedded carbon emissions without the external linkages (carbon purchases and sales) of the target sector. The impact of this extraction on household induced carbon emissions of the rest of the economy is presented by the following equation:

10 $${C}^{H}-{C}^{H\ast }=[\begin{array}{c}{C}_{s}^{H}-{C}_{-s}^{H\ast }\\ {C}_{-s}^{H}-{C}_{-s}^{H\ast }\end{array}]=[\begin{array}{cc}{\tau }_{s} & 0\\ 0 & {\tau }_{-s}\end{array}][\begin{array}{cc}{\psi }_{s,s}-{(I-{A}_{s,s})}^{-1} & {\psi }_{s,-s}\\ {\psi }_{-s,s} & {\psi }_{-s,-s}-{(I-{A}_{-s,-s})}^{-1}\end{array}][\begin{array}{c}{H}_{s}\\ {H}_{-s}\end{array}]$$

The re-modified version of the modified hypothetical extraction to estimate the embedded household (rural and urban) emissions from the intermediate industrial inter and intra-sectoral consumption of goods and services, considering the products emission intensity (intra-sectoral consumption), products upstream supply chain emission intensities (inter-sectoral consumption), production technology and household final demand (the four-dimensional impact) is presented below:

11 $$IC=\mathop{\sum }\limits_{m=1}^{n}{\tau }_{s}{(I-{A}_{s,s})}^{-1}{H}_{sm}$$

12 $$EC=\mathop{\sum }\limits_{m=1}^{n}{\tau }_{-s}{\psi }_{-s,s}{H}_{sm}$$

Where *IC* denotes household induced emissions from intra-sectoral consumption, and *EC* presents household embodied emissions from inter-sectoral (carbon imports) consumption. Similarly, the total household embedded emissions from intra and inter-sectoral carbon consumption can be presented through the following equation:

13 $$TC=IC+EC$$

Where *TC* denotes household induced emissions from the total intermediate industrial consumption of goods and services.

The following formula was used to calculate the CAGR (net emissions change) values over time:

14 $$CAGR={(\frac{Finalyearvalue}{Firstyearvalue})}^{1/No.ofyears}-1$$

## Uncertainty Analysis

‘Uncertainty’ is the lack of ‘certainty’; the certainty is either a ‘logical’ inevitability or a psychological state of confidence irrefutable for the bearer of the confidence^41^. ‘Uncertainty’ estimation quantitatively indicates the ‘quality’ of the results, in the absence of which the results cannot be matched amongst ‘themselves’, with specific references or standards; it ensures the ‘accuracy’ and ‘reliability’ of results^42^. The idea of measurement ‘uncertainty’ is crucial in many scenarios as it informs about the measurement ‘quality’^43^. The ‘GUM (Guide to the Expression of Uncertainty in Measurement)’ specifies the ‘basic framework’ for assessing the measurement uncertainty^44^. The ‘uncertainty’ of the outcomes obtained under an economic model, is an immensely vital issue in ‘economic modeling’^45^. The functional relationship between the ‘measured’ household embedded emissions *C*^*H*^ and the input quantities of sectoral emission intensities (*τ*), Leontief inverse (L), and the household final consumption (H), exhibiting the measurement ‘uncertainty’ of the model final outcome is presented as:

15 $${C}^{H}=f(\tau ,L,H)$$

The functional relationship between the ‘measured’ different type *m* household embedded emissions ${C}_{m}^{H}$ and input variables (quantities) is presented as

16 $${C}_{m}^{H}=f(\tau ,L,{H}_{m})$$

Where *m* represents the Chinese rural and the urban households, while *H*_*m*_ present rural and urban households final consumption. The economic modeling for estimating Chinese rural, urban and combined household embedded carbon emissions from both the industrial consumption and production have been discussed in detail above in sections 2.1 and 2.2 respectively.

As reported above in the introduction section that there exist differences between the sum of provincial energy data and the national energy data released by the energy statistical departments of China. This can lead to uncertainties in measurements estimated based upon the provincial energy data, for example the energy-related provincial industrial emissions of China.

The uncertainties are classified under two major categories by the ‘GUM’, the category ‘A’ and the category ‘B’^46^. The category ‘A’ is based upon some empirical analysis, for example, the ‘standard deviation’ (SD) in a ‘repeatability’ work. In contrast, category ‘B’ uncertainties are based on miscellaneous other sources like expertise, standardization certification etc.^42^. This study uses the category ‘B’ classification of the uncertainties, usually as per the instructions of the ‘GUM’, the category ‘A’ uncertainties are calculated by taking the SD of mean (expected value) acquired from the recurring measurements^42^. In other words, the standard error estimated as the SD of the sample mean. Here, instead of taking the mean of the recurring measurements, the authors have considered the national industrial emission intensities, Leontief inverse values, and the rural and urban final household demand as the expected values, and measured the standard deviations of the aggregated provincial accounts from their expected values.

17 $$\sigma (\tau )=\sqrt{\frac{{\sum }_{i=1}^{n}{({\tau }_{i}-{\tau }_{i}^{E})}^{2}}{N-1}}$$

18 $$\sigma (L)=\sqrt{\frac{{\sum }_{i=1}^{n}{({L}_{i}-{L}_{i}^{E})}^{2}}{N-1}}$$

19 $$\sigma (H)=\sqrt{\frac{{\sum }_{i=1}^{n}{({H}_{i}-{H}_{i}^{E})}^{2}}{N-1}}$$

20 $$\sigma ({H}_{m})=\sqrt{\frac{{\sum }_{m=1}^{k}{\sum }_{i=1}^{n}{({H}_{im}-{H}_{im}^{E})}^{2}}{N-1}}$$

Where $\sigma (\tau ),\sigma (L),\sigma (H)$, and $\sigma ({H}_{m})$ the standard deviation of aggregated provincial industrial emission intensities, Leontief inverse, household final consumption, and different types (rural and urban) household final consumption deviation from their respective expected values from the national statistical data. ${\tau }_{i},\,{L}_{i},\,{H}_{i},$, and *H*_*im*_ represents the aggregated provincial input quantities. While, ${\tau }_{i}^{E},\,{L}_{i}^{E},\,{H}_{i}^{E},\,$and ${H}_{im}^{E}$ represents the national input quantities. *i* denotes different type of industries and *m* denotes different household types. Similarly, the uncertainty of the input quantities of industrial emission intensities $\,r(\tau )$, the Leontief inverse $\,r(L)$, and the total household $\,r(H)$, and household type final consumption $r({H}_{b})$ can be expressed by SD of the sample mean as follows.

21 $$r(\tau )=\frac{\sigma (\tau )}{\sqrt{N}}$$

22 $$r(L)=\frac{\sigma (L)}{\sqrt{N}}$$

23 $$r(H)=\frac{\sigma (H)}{\sqrt{N}}$$

24 $$r({H}_{m})=\frac{\sigma ({H}_{m})}{\sqrt{N}}$$

## Uncertainties Propagation

The ‘GUM’ uncertainty method is grounded on the law of uncertainties propagation. The law simplifies estimations by means of approximations, the ‘uncertainties’ propagation is achieved by considering first order Taylor series approximations. This ‘approximation’ is applicable because the input quantities are large compared to their relative ‘uncertainties’^42^. For simplicity, we present the total household embedded carbon emissions as a function of input quantities *t*_*q*_, where $q\,(q=1,2,3,\cdots w)$ denotes the number of the input quantities, and the household type embedded emissions as a function of the input quantities *t*_*qm*_, the uncertainties propagation of measured variable as a function of input quantities can be presented as below:

25 $${r}_{{C}^{H}}^{2}=\mathop{\sum }\limits_{q=1}^{w}{(\frac{\partial {C}^{H}}{\partial {t}_{q}})}^{2}{r}_{{t}_{q}}^{2}$$

26 $${r}_{{C}_{l}^{H}}^{2}=\mathop{\sum }\limits_{l=1}^{m}\,\mathop{\sum }\limits_{q=1}^{w}{(\frac{\partial {C}_{m}^{H}}{\partial {t}_{q}})}^{2}{r}_{{t}_{ql}}^{2}$$

27 $${r}_{{C}^{H}}=\sqrt{\mathop{\sum }\limits_{q=1}^{w}{(\frac{\partial {C}^{H}}{\partial {t}_{q}})}^{2}{r}_{{t}_{q}}^{2}}$$

28 $${r}_{{C}_{l}^{H}}=\sqrt{\mathop{\sum }\limits_{l=1}^{m}\,\mathop{\sum }\limits_{q=1}^{w}{(\frac{\partial {C}_{m}^{H}}{\partial {t}_{q}})}^{2}{r}_{{t}_{ql}}^{2}}$$

Where ${R}_{{C}^{H}}$ and ${r}_{{C}_{m}^{H}}$ represent the combined standard uncertainty of the measured total household and different household type embedded carbon emissions, ${r}_{{t}_{q}}$ and ${r}_{{t}_{qm}}$ present the standard uncertainties *qth* and the *qmth* input quantity. The partial derivatives in these equations present the coefficients of the sensitivity.

The results obtained from the equation number 27 and 28 relates to a single SD (standard deviation) covering interval. The GUM method for better ‘confidence’ enlarges the interval by assuming a ‘Student’s-t’ distribution. We can use the ‘Welch-Satterthwaite’ method to determine the ‘effective degrees of freedom (df)’^47^.

29 $$d{f}_{eff}({C}^{H})=\frac{{r}_{{C}^{H}}^{4}}{{\sum }_{q=1}^{w}\frac{{r}_{{t}_{q}}^{4}}{d{f}_{{t}_{q}}^{4}}}$$

30 $$d{f}_{eff}({C}_{l}^{H})=\frac{{r}_{{C}^{H}}^{4}}{{\sum }_{l=1}^{m}{\sum }_{q=1}^{w}\frac{{r}_{{t}_{ql}}^{4}}{d{f}_{{t}_{ql}}^{4}}}$$

Where $d{f}_{eff}({C}^{H})$ and $d{f}_{eff}({C}_{l}^{H})$ presents the effective degree of freedom for the total household and household type *m* embedded emissions, $d{f}_{{t}_{q}}^{4}$ and $d{f}_{{t}_{ql}}^{4}$ present the degrees of freedom for the *q th* and the *qm th* input quantity. Now we can estimate the ‘expanded uncertainty’ of the total household embedded and household type embedded emissions by having a product of ‘combined standard uncertainty’ and the coverage factor (*k*)^42,47^.

31 $${R}_{{C}^{H}}=k{r}_{{C}^{H}}$$

32 $${R}_{{C}_{l}^{H}}=\mathop{\sum }\limits_{l=1}^{m}k{r}_{{C}_{l}^{H}}$$

Where ${R}_{{C}^{H}}$ and ${R}_{{C}_{l}^{H}}$ present the ‘expanded uncertainty’ of the total household and household type *l* embedded emissions.

## Monte Carlo Simulation For The Uncertainty Analysis

Monte Carlo simulation (MCS) method has been extensively applied to the input output model related uncertainty measurements^45^. This simulation method has several advantages over the conventional measures of uncertainties^48^. The ‘GUM supplement 1’ provides the general provisions for the ‘propagation’ of uncertainties under this approach^49^. Under MCS, ‘pseudorandom’ numbers are produced using algorithms; afterwards these numbers are imposed to follow a set ‘probability distribution’. If the distribution of these random numbers is normal, the spread of the numbers is set via their specified standard deviation and mean (expected value). The simulation creates a value for each input randomly drawn from it probability distribution, the numbers are produced for all inputs of the function, used to generate ‘single numeric’ estimates as output^44^.

‘GUM supplement 1 (GUM-S1)’ clause 6, for the input quantities p.d.f. (probability density functions), provides additional description on the uncertainties related to the ‘input quantities’^46^. A p.d.f explains the change in a continuous variable’s ‘probability density’ over some allowed ‘range of values’^44^. In other words, the space under a p.d.f between any two ‘points’ of reference is the probability that a random variable lies between these two ‘points’, it’s also known as ‘density’ and ‘density function’^50^. The density function of a ‘normal Gaussian distribution’ is shaped like a bell, with allowed ranges between minus and positive infinity^44^. This study also considers the ‘normal Gaussian distribution’ for the calculation of input uncertainties. This selection is supported by the ‘Central limit theorem’, which states that under some specific conditions, for a large distribution, the sum of the distribution of a random variable is approximately normal^44,50,51^. Additionally, majority of the researchers agree that for ‘Monte Carlo’ simulation of the input-output models, the basic I/O data can be considered as having clear standard deviations, normally distributed, probabilistic errors, and un-correlated traits^27^.

After selecting an appropriate p.d.f, the number of trial simulations should be established, the bigger the number of simulations the bigger is the conjunction of results. The ‘GUM supplement 1’ offers some general guidelines for the selection of appropriate number of trials, which is recommended for having rational presentation of anticipated results^47^.

33 $$B > \frac{{10}^{4}}{1-P}$$

Where *B* represents the recommended number of trials, for example, for a ‘coverage probability’ of 0.68 the number of trials should be at least 31,250. Finally, the tolerance level of an uncertainty is found by articulating the uncertainty as $d\times {10}^{g}$, where d presents an ‘integer’ with equal number of ‘digits’ as the ‘digits’ of the ‘standard uncertainly’ where g is also an ‘integer’^47^.

34 $$\delta =\frac{1}{2}{10}^{g}$$

### Data sources and manupilation

This study uses the 1997, 2000, 2002, 2005, 2007, 2012 and 2015 input-output data from the ‘Chinese Input-output Association’ and from annual data released by ‘National Bureau of Statistics of China’. To remove the effects of price fluxes and convert the data to constant prices; the study employs ‘price index reduction’ technique^52^ and selects 2002 as the base year. The price index related data is from ‘China Price Statistical Yearbook 2013’^53^ and ‘China Price Statistical Yearbook 2016’^54^ respectively. This paper uses the competitive imports assumption, which is normally used to compile input-output tables in the China and the United States^55^. Under this assumption intermediate imports are not distinguished from the domestic intermediate industrial production (demand), i.e. these intermediate imports are domestically converted to final outputs after processing^56^. The gross exports value has been considered for this study. The countries like USA, use the gross exports value without making any adjustments for the import of intermediate industrial goods and services^57^. The input-output data of thirty provinces and municipalities of mainland China comes from ‘China’s economic and social big data research platform’^58^. The provincial carbon emission data is calculated from the energy consumption data of each province. The energy consumption data of each province comes from the National Bureau of statistics of China. Considering the yearly sectoral classification differences in the input-output data, moreover, to correspond with the industrial energy consumption data, the paper reclassifies the industries according to the Chinese national economic industry classification standard GB/T 4754–2017, in to fifteen major industrial sectors. Fossil fuel-based industrial energy consumption data is collected from ‘China Energy Statistical Yearbook 2012’^59^ and ‘China Energy Statistical Yearbook 2016’^60^.

## Supplementary information

Supplementary Information.
