# Supplementary material for: Prospects of industrial consumption embedded final emissions: a revision on Chinese household embodied industrial emissions
Source: Sci Rep. 2020 Feb 4;10:1826. doi: 10.1038/s41598-020-58814-w (PMC7000762; doi:10.1038/s41598-020-58814-w)
Supplement: Supplementary file 1 — Supplementary Information. [file 41598_2020_58814_MOESM1_ESM.pdf]

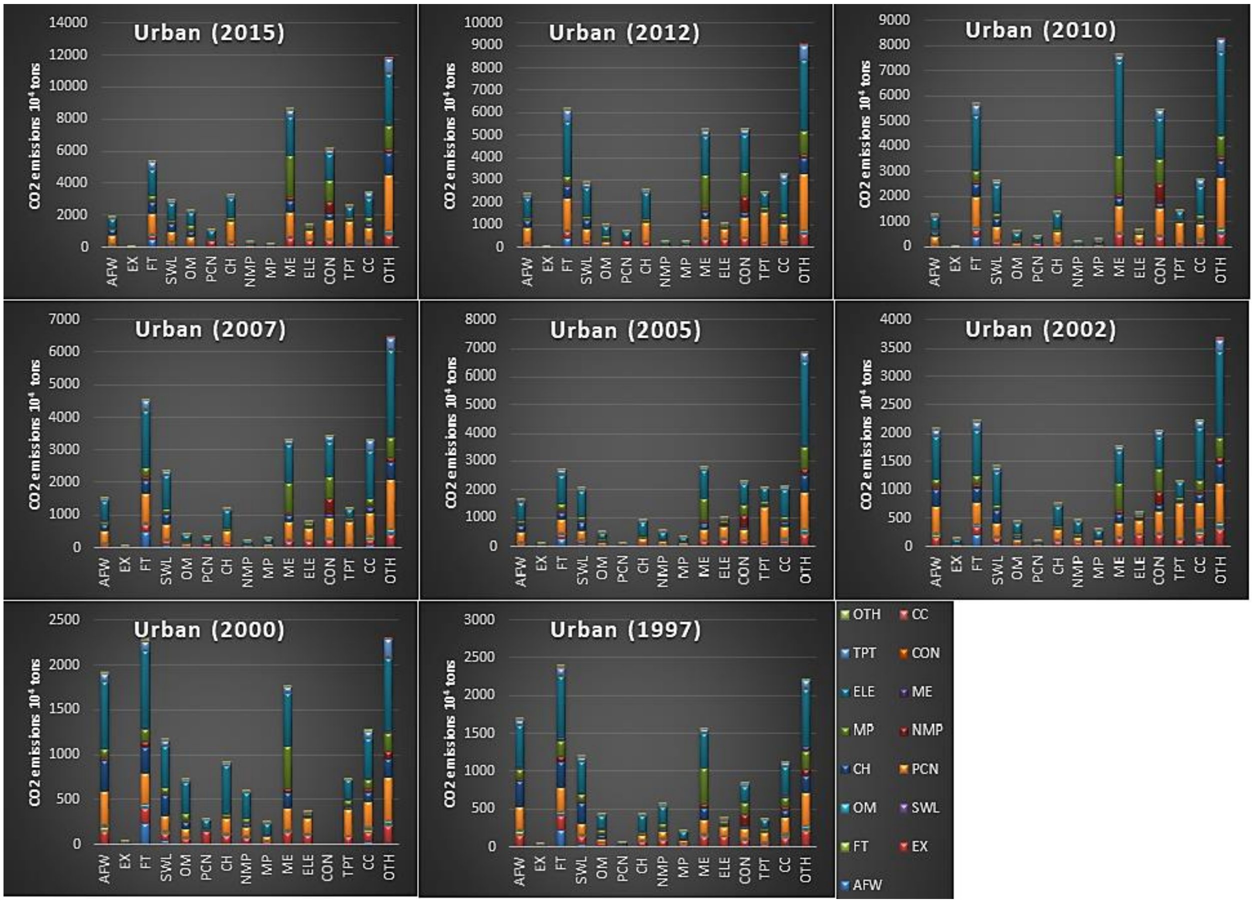

Fig. S1 Further decomposition of urban household embodied temporal emissions from inter-sectoral consumption. The figure represents the urban household induced upstream carbon procurement chain of different sectors.

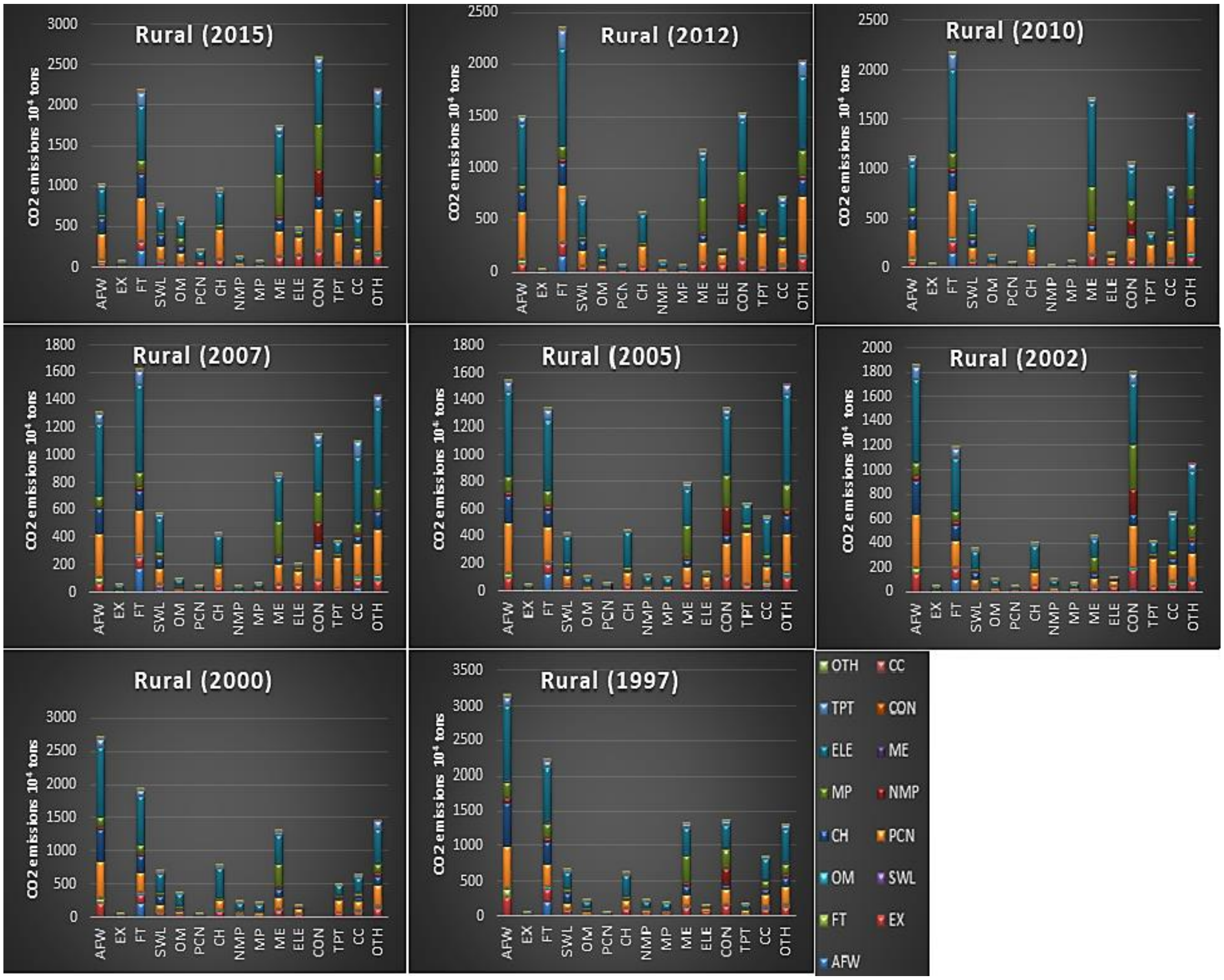

Fig. S2 Further decomposition of rural household embodied temporal emissions from inter-sectoral consumption. The figure represents the rural household induced upstream carbon procurement chain of different sectors.

Table S1 Sectoral codes and temporal carbon emission intensities (tons/10^4 yuan).

Sectors	Codes	Carbon intensity (tons/10,000 yuan)							
		1997	2000	2002	2005	2007	2010	2012	2015
Agriculture, Forestry, Animal Husbandry, Fisheries and Water Conservancy	AFW	0.11	0.08	0.09	0.09	0.09	0.05	0.05	0.06
Extractive industry	EX	1.00	0.84	0.76	0.70	0.55	0.52	0.57	0.48
Food and Tobacco	FT	0.16	0.11	0.11	0.07	0.05	0.04	0.03	0.04
Textile, Sewing and Leather Products	SWL	0.09	0.06	0.06	0.05	0.04	0.04	0.03	0.04
Other manufacturing industries	OM	0.23	0.13	0.11	0.11	0.08	0.08	0.07	0.05
Petroleum processing, Coking and Nuclear fuel industry	PCN	3.33	2.01	3.06	3.16	3.26	3.64	3.81	3.46
Chemical industry	CH	0.69	0.40	0.37	0.29	0.22	0.17	0.17	0.18
Non-metallic Mineral Products	NMP	0.96	0.98	0.96	0.66	0.47	0.39	0.39	0.33
Metal products manufacturing	MP	1.25	0.82	0.69	0.82	0.65	0.60	0.52	0.47
Machinery and Equipment	ME	0.11	0.04	0.03	0.02	0.01	0.01	0.01	0.01
Electricity and Steam, Hot water production and supply	ELE	5.64	3.56	4.14	2.89	2.43	2.10	2.35	1.72
Construction	CON	0.02	0.03	0.02	0.02	0.02	0.01	0.01	0.01
Transport, Post and Telecommunication	TPT	0.44	0.42	0.40	0.43	0.54	0.51	0.11	0.59
Commercial Catering	CC	0.04	0.05	0.04	0.05	0.06	0.06	0.06	0.08
Miscellaneous intangible products	MIP	0.05	0.05	0.04	0.04	0.04	0.05	0.04	0.05

Table S2 Sectoral uncertainty evaluation for the outcome of rural household embedded emissions under the 'GUM LPU' approach.

Sectors	Value	Comb. std. unc.	Expanded uncertainty	Lower Quantile	Upper Quantile
AFW	4.48	5.14	10.28	-5.79	14.76
EX	0.68	1.00	2.01	-1.33	2.69
FT	6.65	7.61	15.21	-8.56	21.86
SWL	1.10	1.84	3.67	-2.58	4.77
OM	0.76	0.96	1.91	-1.16	2.67
PCN	4.30	7.41	14.82	-10.52	19.11
CH	6.37	2.47	4.95	1.42	11.32
NMP	0.87	1.40	2.80	-1.93	3.66
MP	1.14	1.21	2.42	-1.28	3.55
ME	1.26	6.43	12.86	-11.60	14.11
ELE	42.16	11.32	22.64	19.52	64.80
CON	0.25	2.64	5.29	-5.04	5.54
TPT	14.93	5.44	10.87	4.05	25.80
CC	1.44	1.64	3.27	-1.83	4.71
MIP	3.03	3.28	6.56	-3.53	9.58

Table S3 Sectoral uncertainty evaluation for the outcome of rural household embedded emissions under the ‘Monte Carlo’ simulation approach.

Sectors	Value	Comb. std. unc.	Expanded uncertainty	Lower Quantile	Upper Quantile
AFW	4.51	5.16	10.32	-5.76	14.98
EX	0.67	1.02	2.04	-1.35	2.82
FT	6.68	7.64	15.28	-8.42	22.32
SWL	1.08	1.85	3.70	-2.61	4.83
OM	0.76	0.98	1.96	-1.14	2.86
PCN	4.29	7.62	15.23	-10.72	20.50
CH	6.37	2.51	5.03	1.97	12.02
NMP	0.86	1.40	2.80	-1.91	3.69
MP	1.14	1.22	2.43	-1.26	3.62
ME	1.24	6.51	13.02	-11.79	14.48
ELE	42.11	11.40	22.80	21.67	66.87
CON	0.24	2.65	5.30	-5.08	5.55
TPT	14.90	5.56	11.12	5.88	27.92
CC	1.45	1.64	3.29	-1.82	4.80
MIP	3.00	3.56	7.12	-2.95	11.63

Table S4 Sectoral uncertainty evaluation for the outcome of urban household embedded emissions under the 'GUM LPU' approach.

Sectors	Value	Comb. std. unc.	Expanded uncertainty	Lower Quantile	Upper Quantile
AFW	7.00	8.16	16.32	-9.31	23.32
EX	0.44	1.05	2.09	-1.66	2.53
FT	17.39	19.89	39.78	-22.39	57.18
SWL	4.37	7.33	14.66	-10.29	19.04
OM	2.99	3.73	7.47	-4.48	10.45
PCN	45.18	19.26	38.51	6.67	83.69
CH	28.64	10.52	21.05	7.59	49.68
NMP	2.07	1.46	2.92	-0.85	4.98
MP	5.22	6.01	12.01	-6.79	17.23
ME	5.64	28.87	57.74	-52.11	63.38
ELE	210.47	46.60	93.19	117.28	303.66
CON	0.85	9.09	18.18	-17.32	19.03
TPT	62.54	23.37	46.75	15.79	109.28
CC	6.47	7.34	14.68	-8.21	21.14
MIP	13.47	13.45	26.90	-13.43	40.38

Table S5 Sectoral uncertainty evaluation for the outcome of urban household embedded emissions under the 'Monte Carlo' simulation approach.

Sectors	Value	Comb. std. unc.	Expanded uncertainty	Lower Quantile	Upper Quantile
AFW	7.03	8.38	16.77	-9.15	25.25
EX	0.44	1.07	2.13	-1.69	2.66
FT	17.47	19.98	39.96	-22.18	58.54
SWL	4.35	7.38	14.77	-10.38	19.44
OM	2.97	3.79	7.58	-4.43	10.88
PCN	45.24	19.66	39.32	11.43	89.53
CH	28.64	10.67	21.34	9.23	51.93
NMP	2.06	1.47	2.93	-0.86	5.03
MP	5.25	6.03	12.06	-6.74	17.52
ME	5.78	29.31	58.63	-53.13	65.30
ELE	210.63	46.74	93.47	119.86	306.89
CON	0.85	9.13	18.26	-17.43	19.30
TPT	62.66	24.04	48.08	24.01	118.91
CC	6.47	7.35	14.70	-8.19	21.38
MIP	13.50	13.92	27.84	-12.42	44.59

Table S6 Sectoral uncertainty evaluation for the outcome of total household embedded emissions under the ‘GUM LPU’ approach.

Sectors	Value	Comb. std. unc.	Expanded uncertainty	Lower Quantile	Upper Quantile
AFW	11.49	13.26	26.52	-15.04	38.01
EX	1.11	2.05	4.10	-2.99	5.21
FT	24.05	27.50	55.00	-30.95	79.04
SWL	5.47	9.17	18.33	-12.86	23.80
OM	3.75	4.69	9.38	-5.63	13.12
PCN	49.47	26.11	52.22	-2.74	101.69
CH	35.00	12.98	25.95	9.05	60.96
NMP	2.93	2.86	5.71	-2.78	8.64
MP	6.36	7.21	14.43	-8.07	20.79
ME	6.89	35.30	70.60	-63.71	77.49
ELE	252.63	56.39	112.78	139.85	365.41
CON	1.10	11.73	23.46	-22.36	24.56
TPT	77.46	28.81	57.61	19.85	135.08
CC	7.91	8.97	17.94	-10.04	25.85
MIP	16.50	16.67	33.35	-16.85	49.85

Table S7 Sectoral uncertainty evaluation for the outcome of total household embedded emissions under the ‘Monte Carlo’ simulation approach.

Sectors	Value	Comb. std. unc.	Expanded uncertainty	Lower Quantile	Upper Quantile
AFW	11.48	13.53	27.05	-15.11	39.75
EX	1.11	2.08	4.16	-3.02	5.43
FT	24.10	27.72	55.44	-31.00	81.14
SWL	5.48	9.25	18.49	-12.86	24.29
OM	3.74	4.74	9.48	-5.58	13.67
PCN	49.37	26.71	53.43	2.59	109.53
CH	34.98	13.11	26.23	11.28	63.69
NMP	2.94	2.86	5.72	-2.73	8.72
MP	6.35	7.27	14.54	-8.01	21.13
ME	6.93	35.76	71.51	-64.68	79.58
ELE	252.35	56.47	112.94	143.09	369.41
CON	1.14	11.74	23.48	-22.38	24.67
TPT	77.39	29.36	58.73	29.63	146.15
CC	7.88	9.00	18.01	-10.04	26.24
MIP	16.49	17.37	34.75	-15.44	55.97

Table S7 Uncertainty measurement of the different input quantities for the rural household embedded emissions.

Sectors	Parameter	Comb. std. unc.	Sensitivity	Uncertainty cont.
AFW	Emission intensity	0.00	850904.47	5.12
	Leontief inverse	0.19	2.00	0.38
	Final consumption	15854.50	0.00	0.19
EX	Emission intensity	0.00	11895.61	0.13
	Leontief inverse	0.09	0.23	0.02
	Final consumption	5828.34	0.00	1.00
FT	Emission intensity	0.00	1939200.00	7.57
	Leontief inverse	0.27	2.68	0.73
	Final consumption	36535.53	0.00	0.31
SWL	Emission intensity	0.00	429901.28	1.83
	Leontief inverse	0.21	0.49	0.10
	Final consumption	8701.63	0.00	0.05
OM	Emission intensity	0.00	115987.83	0.94
	Leontief inverse	0.16	0.36	0.06
	Final consumption	11734.92	0.00	0.16
PCN	Emission intensity	0.00	11272.49	1.03
	Leontief inverse	0.03	2.69	0.09
	Final consumption	12070.14	0.00	7.34
CH	Emission intensity	0.00	383545.02	2.09
	Leontief inverse	0.55	1.43	0.78
	Final consumption	14352.02	0.00	1.06
NMP	Emission intensity	0.00	22054.65	0.04
	Leontief inverse	0.11	0.47	0.05
	Final consumption	19543.53	0.00	1.40
MP	Emission intensity	0.00	21804.46	0.08
	Leontief inverse	0.22	0.36	0.08
	Final consumption	7372.85	0.00	1.20
ME	Emission intensity	0.00	1333200.00	6.43
	Leontief inverse	0.66	0.28	0.18
	Final consumption	7079.74	0.00	0.03
ELE	Emission intensity	0.00	179233.97	8.88
	Leontief inverse	0.20	14.23	2.88
	Final consumption	9195.09	0.00	6.41
CON	Emission intensity	0.00	231754.80	2.64
	Leontief inverse	0.05	0.20	0.01
	Final consumption	4699.72	0.00	0.01
TPT	Emission intensity	0.00	1421700.00	3.03
	Leontief inverse	1.02	3.06	3.12
	Final consumption	63750.39	0.00	3.27
CC	Emission intensity	0.00	236956.59	1.63
	Leontief inverse	0.00	0.93	0.00
	Final consumption	17648.30	0.00	0.17
MIP	Emission intensity	0.00	703567.65	2.91
	Leontief inverse	0.12	1.29	0.15
	Final consumption	150075.56	0.00	1.51

Table S8 Uncertainty measurement of the different input quantities for the urban household embedded emissions.

Sectors	Parameter	Comb. std. unc.	Sensitivity	Uncertainty cont.
AFW	Emission intensity	0.00	1328600.00	8.00
	Leontief inverse	0.19	3.12	0.59
	Final consumption	126376.42	0.00	1.50
EX	Emission intensity	0.00	7654.75	0.09
	Leontief inverse	0.09	0.15	0.01
	Final consumption	6099.79	0.00	1.04
FT	Emission intensity	0.00	5071000.00	19.78
	Leontief inverse	0.27	7.00	1.92
	Final consumption	96574.19	0.00	0.82
SWL	Emission intensity	0.00	1715200.00	7.31
	Leontief inverse	0.21	1.96	0.41
	Final consumption	55210.69	0.00	0.31
OM	Emission intensity	0.00	456709.74	3.71
	Leontief inverse	0.16	1.40	0.22
	Final consumption	28202.60	0.00	0.40
PCN	Emission intensity	0.00	118575.78	10.80
	Leontief inverse	0.03	28.32	0.91
	Final consumption	26181.13	0.00	15.92
CH	Emission intensity	0.00	1725100.00	9.42
	Leontief inverse	0.55	6.43	3.51
	Final consumption	42148.58	0.00	3.12
NMP	Emission intensity	0.00	52686.69	0.09
	Leontief inverse	0.11	1.13	0.12
	Final consumption	20292.58	0.00	1.45
MP	Emission intensity	0.00	100261.32	0.36
	Leontief inverse	0.22	1.67	0.37
	Final consumption	36671.33	0.00	5.98
ME	Emission intensity	0.00	5987800.00	28.86
	Leontief inverse	0.66	1.24	0.81
	Final consumption	16288.48	0.00	0.07
ELE	Emission intensity	0.00	894731.11	44.32
	Leontief inverse	0.20	71.05	14.36
	Final consumption	1251.58	0.00	0.87
CON	Emission intensity	0.00	796575.49	9.09
	Leontief inverse	0.05	0.67	0.03
	Final consumption	56031.28	0.00	0.08
TPT	Emission intensity	0.00	5955700.00	12.69
	Leontief inverse	1.02	12.81	13.06
	Final consumption	286011.30	0.00	14.66
CC	Emission intensity	0.00	1065600.00	7.31
	Leontief inverse	0.00	4.19	0.00
	Final consumption	63253.52	0.00	0.59
MIP	Emission intensity	0.00	3133400.00	12.94
	Leontief inverse	0.12	5.75	0.68
	Final consumption	358834.88	0.00	3.62

Table S9 Uncertainty measurement of the different input quantities for the total household embedded emissions.

	Parameter	Comb. std. unc.	Sensitivity	Uncertainty cont.
AFW	Emission intensity	0.00	2179600.00	13.12
	Leontief inverse	0.19	5.11	0.96
	Final consumption	142230.92	0.00	1.68
EX	Emission intensity	0.00	19550.36	0.22
	Leontief inverse	0.09	0.37	0.03
	Final consumption	11928.13	0.00	2.04
FT	Emission intensity	0.00	7010200.00	27.35
	Leontief inverse	0.27	9.67	2.65
	Final consumption	133109.72	0.00	1.14
SWL	Emission intensity	0.00	2145100.00	9.15
	Leontief inverse	0.21	2.45	0.51
	Final consumption	63912.32	0.00	0.36
OM	Emission intensity	0.00	572697.57	4.65
	Leontief inverse	0.16	1.75	0.27
	Final consumption	39937.53	0.00	0.56
PCN	Emission intensity	0.00	129848.27	11.83
	Leontief inverse	0.03	31.01	1.00
	Final consumption	38251.27	0.00	23.25
CH	Emission intensity	0.00	2108700.00	11.51
	Leontief inverse	0.55	7.86	4.30
	Final consumption	56500.60	0.00	4.18
NMP	Emission intensity	0.00	74741.34	0.12
	Leontief inverse	0.11	1.61	0.17
	Final consumption	39836.11	0.00	2.85
MP	Emission intensity	0.00	122065.78	0.43
	Leontief inverse	0.22	2.03	0.45
	Final consumption	44044.18	0.00	7.19
ME	Emission intensity	0.00	7321000.00	35.29
	Leontief inverse	0.66	1.51	1.00
	Final consumption	23368.22	0.00	0.10
ELE	Emission intensity	0.00	1074000.00	53.20
	Leontief inverse	0.20	85.28	17.23
	Final consumption	10446.67	0.00	7.28
CON	Emission intensity	0.00	1028300.00	11.73
	Leontief inverse	0.05	0.86	0.04
	Final consumption	60730.99	0.00	0.08
TPT	Emission intensity	0.00	7377400.00	15.72
	Leontief inverse	1.02	15.87	16.17
	Final consumption	349761.69	0.00	17.92
CC	Emission intensity	0.00	1302600.00	8.94
	Leontief inverse	0.00	5.12	0.00
	Final consumption	80901.82	0.00	0.76
MIP	Emission intensity	0.00	3836900.00	15.84
	Leontief inverse	0.12	7.04	0.84
	Final consumption	508910.43	0.00	5.13
